# Neuropilin-1 deficiency in vascular smooth muscle cells is associated with hereditary hemorrhagic telangiectasia arteriovenous malformations

**DOI:** 10.1172/jci.insight.155565

**Published:** 2022-05-09

**Authors:** Sreenivasulu Kilari, Ying Wang, Avishek Singh, Rondell P. Graham, Vivek Iyer, Scott M. Thompson, Michael S. Torbenson, Debabrata Mukhopadhyay, Sanjay Misra

**Affiliations:** 1 Vascular and Interventional Radiology Translational laboratory, Division of Vascular and Interventional Radiology, Department of Radiology,; 2 Department of Cardiovascular Medicine,; 3 Department of Biochemistry and Molecular Biology,; 4 Department of Laboratory Medicine and Pathology, and; 5 Mayo Clinic Hereditary Hemorrhagic Telangiectasia Center of Excellence, Mayo Clinic, Rochester, Minnesota, USA.; 6 Department of Biochemistry and Molecular Biology, College of Medicine and Science, Mayo Clinic, Jacksonville, Florida, USA.

**Keywords:** Vascular Biology, Apoptosis, Cardiovascular disease, Genetic diseases

## Abstract

Patients with hereditary hemorrhagic telangiectasia (HHT) have arteriovenous malformations (AVMs) with genetic mutations involving the activin-A receptor like type 1 (*ACVRL1* or *ALK1*) and endoglin (*ENG*). Recent studies have shown that Neuropilin-1 (NRP-1) inhibits ALK1. We investigated the expression of NRP-1 in livers of patients with HHT and found that there was a significant reduction in NRP-1 in perivascular smooth muscle cells (SMCs). We used *Nrp1^SM22KO^* mice (*Nrp1* was ablated in SMCs) and found hemorrhage, increased immune cell infiltration with a decrease in SMCs, and pericyte lining in lungs and liver in adult mice. Histologic examination revealed lung arteriovenous fistulas (AVFs) with enlarged liver vessels. Evaluation of the retina vessels at P5 from *Nrp1^SM22KO^* mice demonstrated dilated capillaries with a reduction of pericytes. In inflow artery of surgical AVFs from the *Nrp1^SM22KO^* versus WT mice*,* there was a significant decrease in *Tgfb1*, *Eng*, and *Alk1* expression and phosphorylated SMAD1/5/8 (pSMAD1/5/8), with an increase in apoptosis. TGF-β1–stimulated aortic SMCs from *Nrp1^SM22KO^* versus WT mice have decreased pSMAD1/5/8 and increased apoptosis. Coimmunoprecipitation experiments revealed that NRP-1 interacts with ALK1 and ENG in SMCs. In summary, NRP-1 deletion in SMCs leads to reduced ALK1, ENG, and pSMAD1/5/8 signaling and reduced cell death associated with AVM formation.

## Graphical abstract

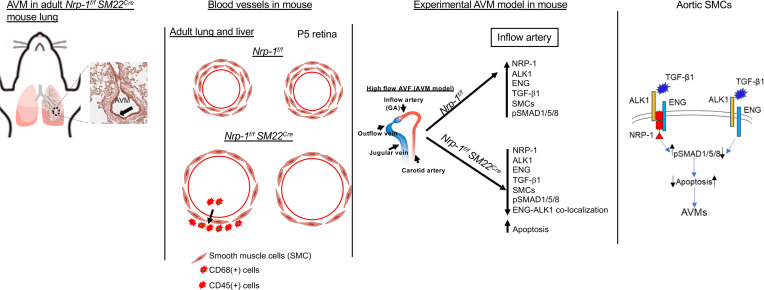

## Introduction

Hereditary hemorrhagic telangiectasia (HHT) is an autosomal dominant multisystemic vascular disorder characterized by the presence of arteriovenous malformations (AVMs) in multiple organs, including the brain, lung, liver, gastrointestinal tract, mucus membrane, and the skin (1–4). AVMs are an aberrant connection between the artery to the vein with an abnormal intervening capillary bed. They have high pressure and low flow through the capillary bed. Arteriovenous fistulas (AVFs) are an aberrant connection of the artery to vein directly without an intervening capillary bed (5). They have high flow with high pressure. Cavernous angiomas are postcapillary, venule malformations with low pressure and low flow. AVMs may have associated aneurysms due to high pressure and to shear stress and fragility of the vessels that can lead to rupture and subsequent hemorrhage (6).

The exact molecular mechanisms of the formation of AVMs are not well understood but are thought to be related to impaired cell signaling, apoptosis, cell-to-cell interactions, and genetic abnormalities (1, 6, 7). High blood flow in the inflow artery and outflow vein of the AVM occurs, and it is accepted that these hemodynamic changes may cause the AVMs to increase in size and rupture (6). The AVM vessel walls are friable because of a thin wall that possesses few smooth muscle cells (SMCs), suggesting that SMCs may play a pivotal role in the pathogenesis of AVMs (8–10). Surgical models have been created to study hemodynamic changes associated with AVMs, but the contribution of SMCs to the integrity of the vascular wall and subsequent remodeling in the pathogenesis of AVMs has not been well studied (11).

The majority of genetic mutations seen in patients with HHT involve the activin-A receptor like type 1 (*ACVRL1* or *ALK1*) and endoglin (*ENG*), which are TGF-β family cell surface receptors (5, 12). Other gene mutations have been reported, including suppressor of mothers against decapentaplegic 4 (*SMAD4*) and bone morphogenic protein 9 (*BMP9*). The signaling cues responsible for the formation of AVM in patients remains unknown (13). Transgenic animal models with ablation of *Alk1*, *Eng*, and *Smad4* genes have been generated, and they exhibit AVM formation in adult animals (14–17). For example, histologic evaluation of blood vessels from *Alk1*- or *Eng*-deficient mice have aberrant vascular SMC recruitment, which recapitulates the clinical histologic features of HHT (15, 18–20). Moreover, reduction of circulating TGF-β1 in patients with HHT-1 has been attributed to the reduction of TGF-β production by endothelial cells (ECs) due to reduced *ENG* levels, and this reduction affects the recruitment of SMCs to the vessel wall resulting in leaky and fragile vessels (21, 22). However, the role of TGF-β1/ALK1 or TGF-β1/ENG signaling in vascular SMCs in vascular remodeling and vascular malformations has not been defined.

A recent study showed that Neuropilin-1 (NRP-1) inhibits ALK1 signaling in tip cells during vascular sprouting, as assessed using a postnatal retinal angiogenesis model in the mouse (23). NRP-1 can function as both a receptor or a coreceptor and is involved in multiple signaling pathways implicated in the pathogenesis of neurovascular systems (12, 24–26). Studies have demonstrated that genetic ablation of NRP-1 results in very diverse phenotypes, depending on the cell from which *Nrp1* has been selectively deleted. *Nrp1* deletion in ECs (27) is known to be lethal during embryonic development at E11.5 because of the presence of vascular defects resulting in hemorrhage (28). However, *Nrp1* deletion in SMCs and cardiomyocytes are viable, and these animals present with decreased blood pressure, cardiac hypertrophy, and infiltration of perivascular inflammatory cells into the lungs (28). Another study reported that NRP-1 is involved in SMC differentiation via platelet-derived growth factor (PDGF) signaling (29).

The goal of the present study was to investigate the role of AVM formation caused by *Nrp1* deletion in SMCs using in vitro and in vivo experimental models. We first analyzed whether there were differences in NRP-1 expression by performing immunostaining in livers removed from patients with HHT with ALK1 mutation compared with controls. Next, we utilized a Cre-Lox mouse line in which *Nrp1* was selectively deleted in cells expressing the SM22α gene (*Nrp1^SM22KO^*) to investigate the role of NRP-1 in SMCs in AVM development by assessing the postnatal retinal vessels, adult lungs, and livers. To study the vascular remodeling seen in patients with HHT because of an AVF, we created surgical AVFs by connecting the carotid artery to the ipsilateral jugular vein (11, 30). We determined the changes in NRP-1, ALK1, ENG, TGF-β1, TNF-α, and BMP9 expression in the artery supplying the AVF and in aortic SMCs isolated from *Nrp1^SM22KO^* mice for caspase-3/7 activity and proliferation. Finally, we performed coimmunoprecipitation experiments to determine binding interactions between NRP-1 with ALK1 and ENG. Taken collectively, the results from the present study indicate that NRP-1 deletion in SMC leads to a decrease in ALK1/ENG signaling and to a decrease in pSMAD1/5/8 in SMCs contributing to formation of AVMs associated with HHT (ALK1) phenotype.

## Results

### There is decreased NRP-1 staining in perivascular SMCs in the livers from patients with HHT with ALK1 mutation.

**Figure 1:**
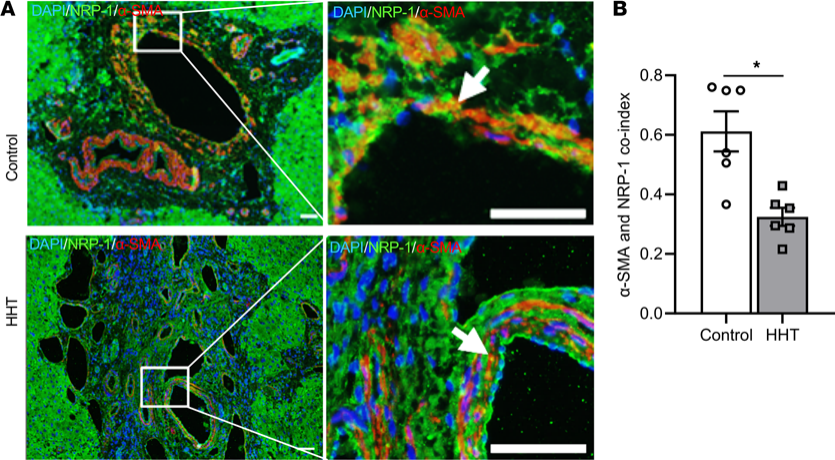
NRP-1 expression in liver sections from patients with HHT and controls. (**A**) Coimmunostaining for NRP-1 (green), α-SMA staining (red), and DAPI-stained (blue) nucleus in representative images. The part of the images enclosed with boxes in the left panel were digitally enlarged and shown as right panel. Arrows indicate cells that are positive for both α-SMA and NRP-1. Multiple vacuoles with minimal α-SMA^+^ cells can be found in HHT patient liver sections (lower panel). All images were captured at 10× magnification using a Zeiss Axio imager M2 equipped with an Axiocam 503 camera. Scale bar: 50 μm. (**B**) The overlapped green and red stain intensity index was determined using Fiji-ImageJ software with JACoP plugin. Data are shown as mean ± SEM of *n* = 6. Nonparametric Mann-Whitney *U* test was performed. *P < 0.05.

We performed coimmunostaining for both NRP-1 and α-SMA in human liver sections. We observed that there was NRP-1^+^ staining in blood vessels (Figure 1 and Supplemental Figure 1; supplemental material available online with this article; https://doi.org/10.1172/jci.insight.155565DS1) and hepatocytes as shown previously (31, 32). There is a significant reduction of cells staining positive for both NRP-1 and α-SMA in liver tissue sections of patients with HHT compared with controls (HHT: 0.32 ± 0.03, control: 0.61 ± 0.068, average decrease: 47.54%, *P* = 0.0087; Figure 1 and Supplemental Figure 1).

### Nrp1 deletion is specific to SM22^+^ cells in Nrp1^SM22KO^ mice.

NRP-1 deletion in SMCs of *Nrp1^SM22KO^* mice was validated by coimmunostaining in mouse tissue sections and in isolated SMCs. We used SM22α and CD31 antibodies to label SMCs and ECs. Coimmunostaining showed that SM22α and CD31 specifically labeled the SMCs and EC, respectively (Supplemental Figure 2). We next determined if NRP-1 was deleted in SM22α^+^ cells of *Nrp1^SM22KO^* mice by performing immunostaining in lung tissue sections. We observed no NRP-1 expression in cells positive for SM22α in *Nrp1^SM22KO^* mouse lungs compared with WT sex-matched littermates (Supplemental Figure 3). Next, we performed Western blot analysis for NRP-1 in SMCs isolated from the aorta, and it revealed that NRP-1 protein was not detected *Nrp1^SM22KO^* mice compared with WT sex-matched littermates. However, in pulmonary ECs from *Nrp1^SM22KO^* mice compared with WT sex-matched littermates, we detected NRP-1 expression; furthermore, there was no change in NRP-1 protein levels (Supplemental Figure 4), demonstrating that NRP-1 was specifically deleted in SMCs.

### Nrp1^SM22KO^ mice have pulmonary AVFs with dilated blood vessels in the lung and liver.

**Figure 2:**
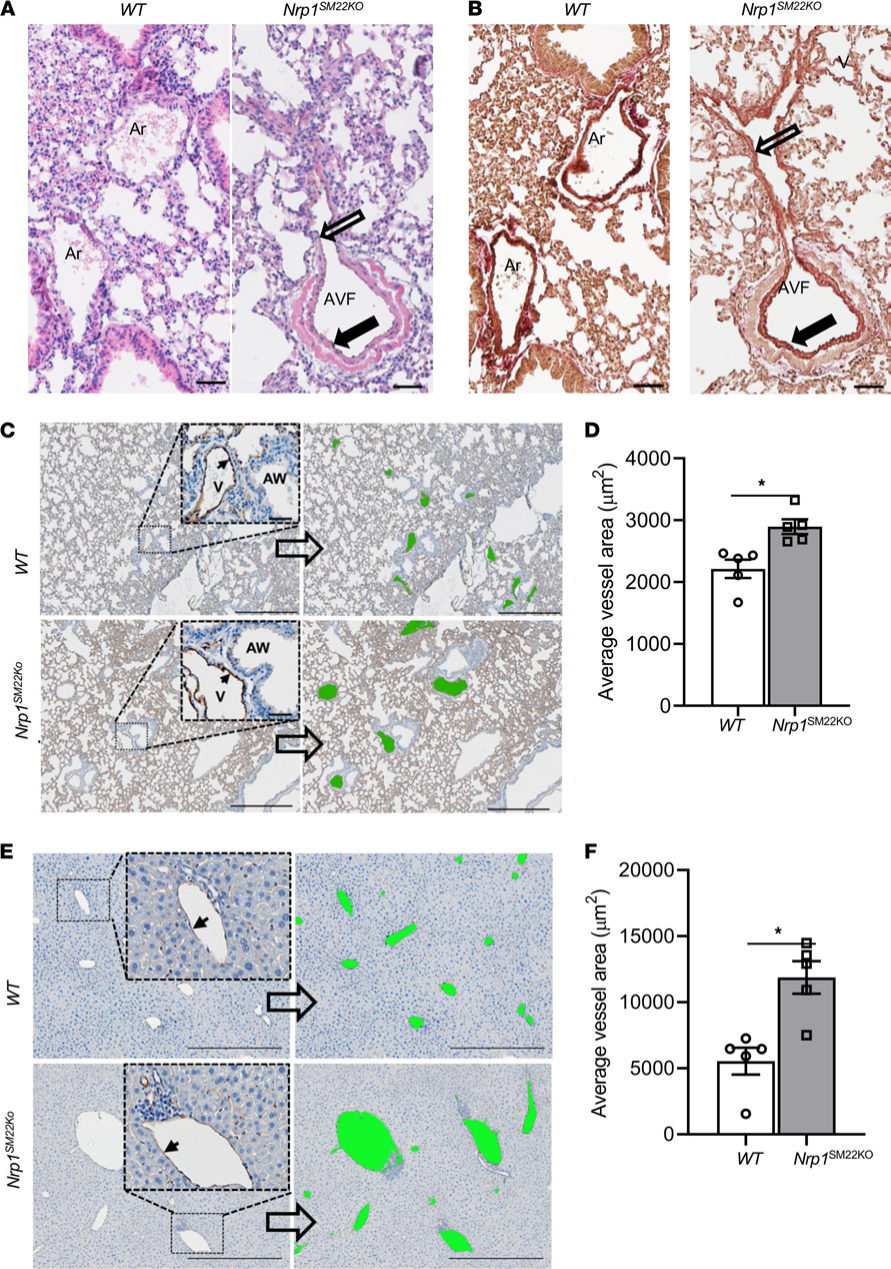
Direct connection of artery to vein in the lung and dilated vessels in lung and liver of *Nrp1^SM22KO^* mice. (**A**) Lung tissue sections were stained for H&E, which shows heterogenic thickness of the vessel (open and closed arrows) in *Nrp1^SM22KO^* animals. (**B**) Verhoeff–Van Gieson–stained sections from *Nrp1^fl/fl^* (WT) and *Nrp1^fl/fl^/SM22α^Cre+^* (*Nrp1^SM22KO^*) mice were used to distinguish the artery (closed arrow) and vein (open arrow). Ar, artery; V, vein; AW, airway in lungs. Scale bar: 50 μm. (**C**–**F**) vascular cross-sectional area was determined in CD31-stained lung (**C** and **D**) liver tissue sections (**E** and **F**). All images were captured at 10× magnification using a Zeiss Axio imager M2 equipped with an Axiocam 503 camera and a motorized stage. Scale bar: 500 μm. The area enclosed with the dotted line box was digitally enlarged to show as inset panel. The solid black arrows indicate CD31^+^ cells lining the blood vessels. Vessels were identified manually and shaded in green, as shown in right panels. The average vessel area in lung (**C**) and liver (**E**) was measured using Fiji-ImageJ software, and the average cross-sectional area in lung (**D**) and in liver (**F**) were shown as bar graphs. Data are shown as mean ± SEM of *n* = 5. There is a significant increase in average cross-sectional area of vessels in lung (**D**) and liver (**E**) from *Nrp1^SM22KO^* mice compared with WT animals. Nonparametric Mann-Whitney *U* test was performed. *P < 0.05.

Examination of H & E-stained lung sections (Figure 2A) and Verhoeff–Van Gieson (VVG) staining (Figure 2B and Supplemental Figure 5) was performed to delineate the pulmonary artery that has both an internal and external elastic lamina compared with pulmonary veins that have internal elastic laminae. We observed that there was AVF formation in *Nrp1^SM22KO^* mouse lungs compared with WT sex-matched littermates (Figure 2B). However, we did not see this in the liver (Supplemental Figure 5C). We performed CD31 staining in the lung and liver tissue sections to determine the cross-sectional area of blood vessels. Semiquantitative analysis demonstrated a significant enlargement of CD31^+^ vessels in the lungs (*Nrp1^SM22KO^*: 2896 ± 119.9 μm^2^, WT: 2213 ± 149 μm^2^, average increase: 130.86%, *P* = 0.0079; Figure 2, C and D) of *Nrp1^SM22KO^* mice compared with WT sex-matched littermates. Semiquantitative analysis demonstrated a significant enlargement of CD31^+^ vessels in *Nrp1^SM22KO^* mice compared with WT sex-matched littermate (*Nrp1^SM22KO^*: 11875 ± 1238 μm^2^, WT: 5535 ± 1018 μm^2^, average increase: 214.54%, *P* = 0.0079; Figure 2, E and F).

### Nrp1^SM22KO^ mice have presence of hemorrhage, increased immune cell infiltration with decrease in SMCs, and pericyte lining in lungs and liver.

**Figure 3:**
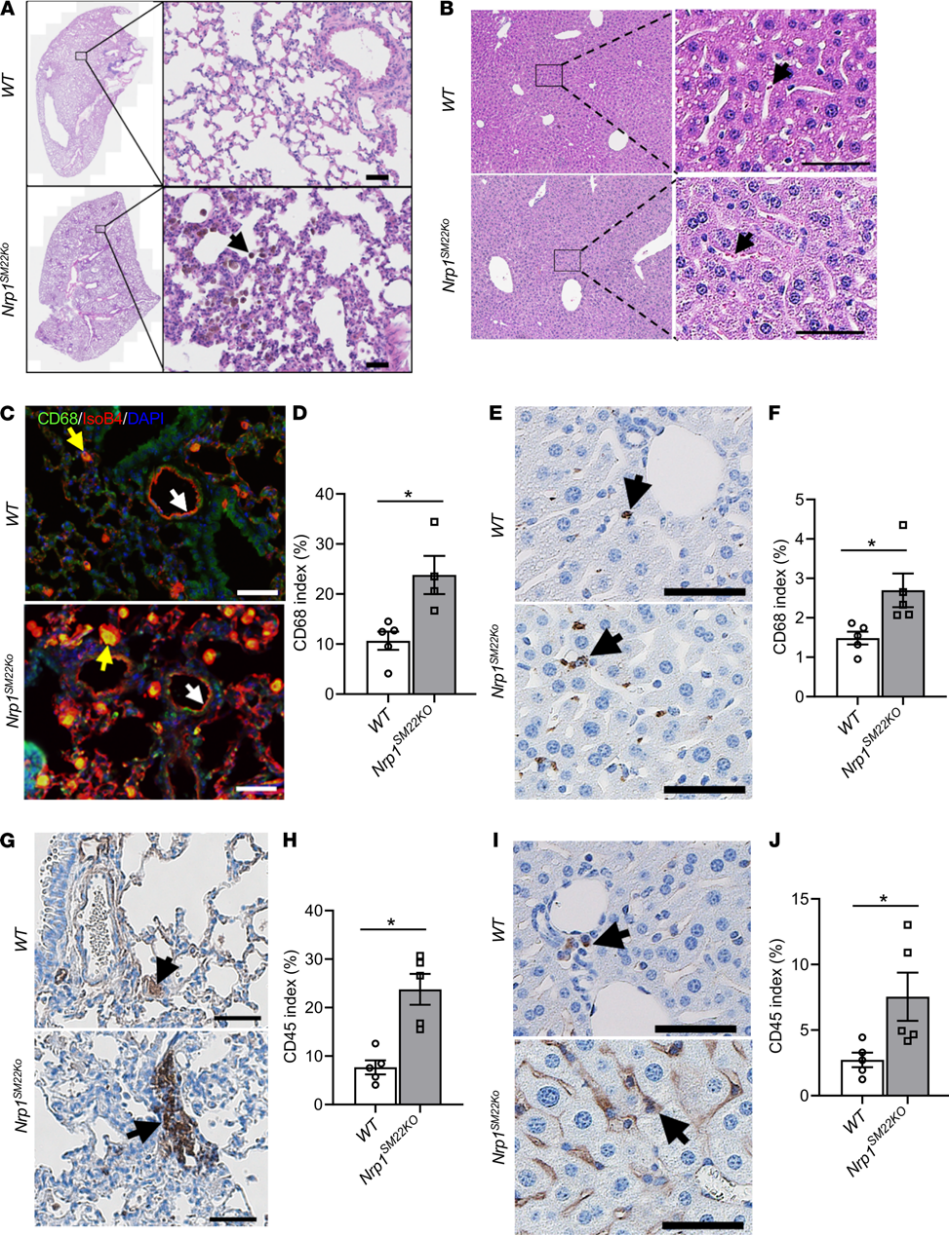
Augmented immune cell infiltration in lung and liver of *Nrp1^SM22KO^* mice. (**A** and **B**) H&E staining of lung (**A**) and liver (**B**) tissue sections from *Nrp1^fl/fl^* (WT) and *Nrp1^fl/fl^/SM22α^Cre+^* (*Nrp1^SM22KO^*) mice. All images were captured at 10× magnification using a Zeiss Axio imager M2 equipped with an Axiocam 503 camera and a motorized stage. Arrows indicate infiltrated cells in the interstitial compartments of lung and liver tissue. The areas enclosed by boxes in representative images are digitally enlarged and shown as separate panels. (**C**) Coimmunostaining for CD68 (green) and isolectin-B4 (red)staining and DAPI-stained (blue) nuclei. The yellow arrows point to cells positive for both CD68 and isolectin-B4, and white arrows point to isolectin positive endothelium. (**D**) Fluorescence intensity quantification of CD68^+^ green stain in **C**. (**E**, **G**, and **I**) Chromogen stain for CD68 in liver (**E**) and CD45 in lung (**G**) and liver (**I**). The black arrows point to brown cells positive for CD68 in liver (**E**) and CD45 in lung (**G**) and liver (**I**). Scale bar: 50 μm. The brown stain intensity was measured using Zen Pro image analysis software (Zeiss). (**F**, **H**, and **J**) The percentage of stained area over total tissue area was presented as stain index of CD68 in liver (**F**) and CD45 index in lung (**H**) and liver (**J**). Data are shown as mean ± SEM of *n* = 5. Nonparametric Mann-Whitney *U* test was performed. *P < 0.05.

H&E-stained lung (Figure 3A) and liver (Figure 3B) sections demonstrate areas of hemorrhage. Carstairs’ and Masson’s trichrome staining of the lung confirms the presence of extravascular blood cells (Supplemental Figure 6, A and B). We investigated the presence of macrophages by performing 594 Alexa Fluor–conjugated isolectin-B4 along with CD68 costaining to visualize the vasculature in mouse lungs. There was an increase in cells staining positive for CD68 in the lung alveoli of *Nrp1^SM22KO^* mice compared with WT sex-matched littermate (*Nrp1^SM22KO^*: 23.80 ± 3.81, WT: 10.67 ± 1.82, average increase: 223%, *P* = 0.016; Figure 3, C and D, and Supplemental Figure 7). In the liver, there was a significant increase in cells staining positive for CD68 in the interstitial compartment of *Nrp1^SM22KO^* mice compared with WT sex-matched littermate (*Nrp1^SM22KO^*: 2.70 ± 0.43, WT: 1.48 ± 0.16, average increase: 182%, P = 0.0079; Figure 3, E and F). We next performed CD45 staining to assess leukocyte infiltration in lung and liver tissues. There was a significant increase in CD45^+^ cells in the perivascular compartments of the lung (*Nrp1^SM22KO^:* 23.75 ± 3.19, WT: 7.67 ± 1.44, average increase: 309.65%, *P* = 0.0079; Figure 3, G and H) and interstitial compartments of the liver (*Nrp1^SM22KO^:* 7.54 ± 1.84, WT: 2.73 ± 0.55, average increase: 276%, *P* = 0.016; Figure 3, I and J) from *Nrp1^SM22KO^* mice compared with WT sex-matched littermates.

**Figure 4:**
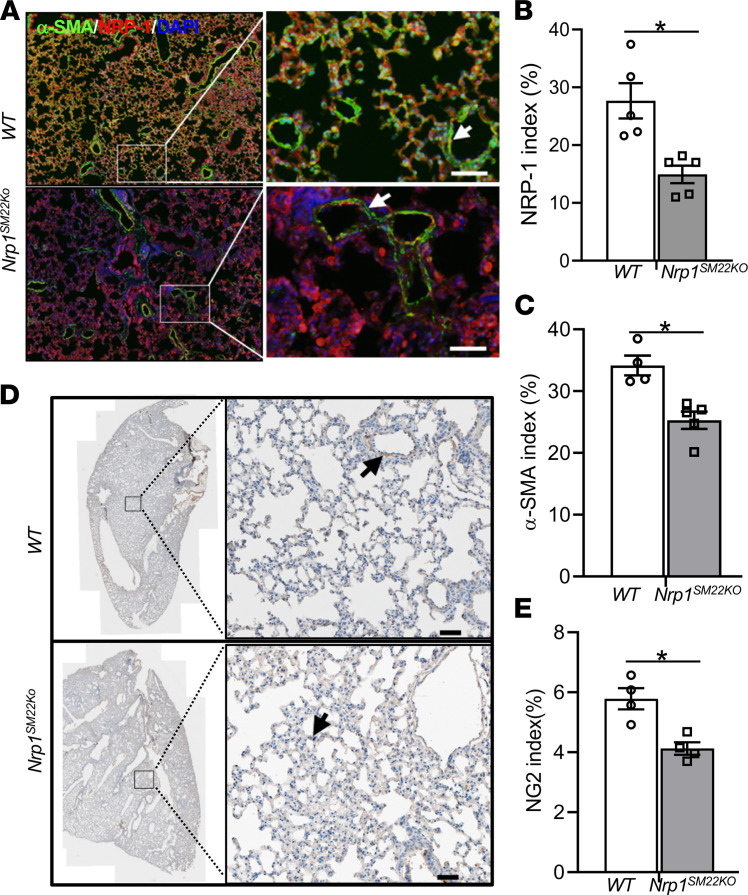
*Nrp1^SM22KO^* mice have decreased smooth muscle cells and pericytes in adult lungs. Coimmunostaining for (**A**–**C**) α-SMA (green), NRP-1(red), and nuclei (blue) stained with DAPI. (**D**) Chromogen staining for NG2^+^ pericyte lining in lung vasculature was performed. White arrows point to α-SMA^+^ lung vasculature in **A** and black arrows in **E** point to NG2^+^ brown pericytes. All images were captured at 10× magnification using a Zeiss Axio imager M2 equipped with an Axiocam 503 camera and a motorized stage. Scale bar: 50 μm. Fluorescence intensity quantification of red NRP-1^+^ (**B**) and green α-SMA^+^ (**C**) present in **A**. (**E**) NG2^+^ brown chromogen stain intensity in **D** was measured using Zen Pro image analysis software (Zeiss) and presented as bar graphs. There is a minimum green α-SMA and brown NG2 staining in the lung alveoli of *Nrp1^SM22KO^* mice compared with WT mice. Data are shown as mean ± SEM of *n* = 4 or 5 animals. Nonparametric Mann-Whitney *U* test was performed. *P < 0.05.

We next determined the smooth muscle density in the lungs and livers of these animals. There was a significant decrease in the α-SMA staining in the alveolar septa of lungs of *Nrp1^SM22KO^* mice compared with WT sex-matched littermates (*Nrp1^SM22KO^*: 25.29 ± 1.39, WT: 34.15 ± 1.59, average decrease: 26%, *P* = 0.016; Figure 4C, and Supplemental Figure 8). As expected, NRP-1 staining was decreased in *Nrp1^SM22KO^* lungs compared with WT sex-matched littermate (*Nrp1^SM22KO^*: 14.94 ± 1.51, WT: 27.67 ± 3.042, average decrease: 46%, *P* = 0.0079; Figure 4, A and B). In addition, there was a significant decrease in the pericyte index as assessed by NG2 staining in the lungs from *Nrp1^SM22KO^* mice compared with WT sex-matched littermate (*Nrp1^SM22KO^*: 4.12 ± 0.21, WT: 5.78 ± 0.35, average decrease: 29%, *P* = 0.029; Figure 4, D and E).

### Nrp1^SM22KO^ mice have dilated capillaries and decreased pericytes in P5 retinal vasculature.

**Figure 5:**
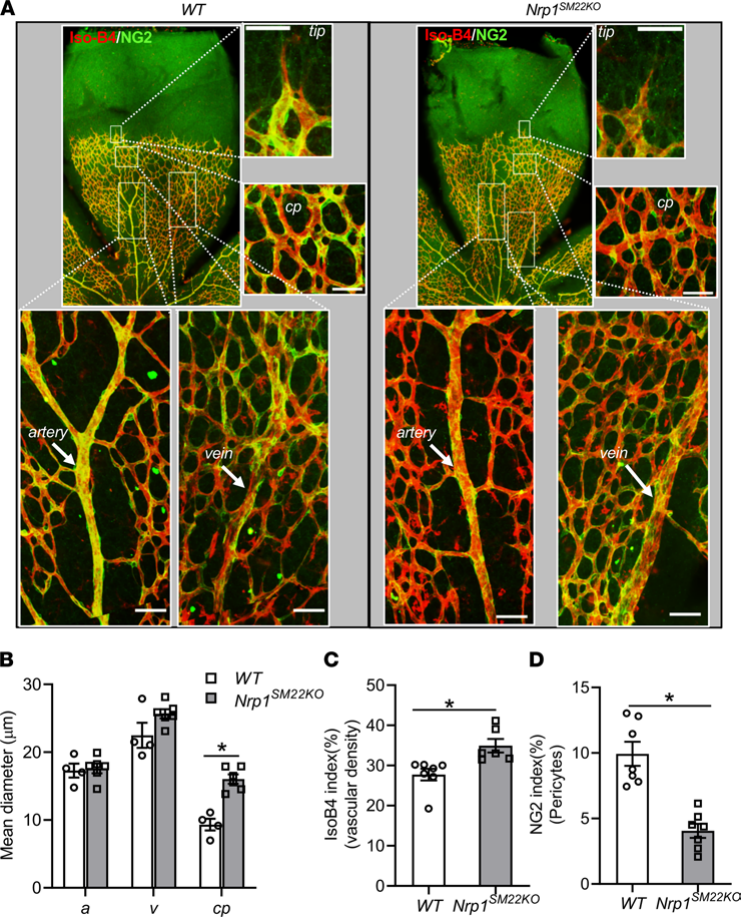
Smooth muscle cell *Nrp1* deletion results in increase vascular diameter and density with a decrease in pericyte lining in the retinal vasculature. (**A**) P5 retinas from *Nrp1^fl/fl^* (WT) and *Nrp1^fl/fl^/SM22α^Cre+^* (*Nrp1^SM22KO^*) mouse pups were stained for red isolectin-B4 (IsoB4) and NG2 for pericytes. The areas enclosed by boxes in representative images are digitally enlarged and shown as separate panels. Scale bar: 50 μm. (**B**) Vascular diameter was measured using Zen Pro software. Data are shown as mean diameter of artery (*a*), vein (*v*), and capillaries (*cp*). (**C** and **D**) Fluorescence intensity quantification of red IsoB4^+^ vasculature and green NG2^+^ (**D**) cells was performed using NIH ImageJ software. All images were captured at 10× objective and digitally enlarged to show as separate panels. Data are shown as mean ± SEM of *n* = 4–7 retinas. Nonparametric Mann-Whitney *U* test was performed. *P < 0.05.

Although previous results demonstrated that *Nrp1* deletion in ECs leads to inhibition of sprouting angiogenesis in the mouse retina (23), our results show that *Nrp1* deletion in SMCs did not affect sprouting angiogenesis (Figure 5A and Supplemental Figure 9). However, there were enlarged capillary plexuses observed in *Nrp1^SM22KO^* mouse retinas compared with WT sex-matched littermate (*Nrp1^SM22KO^*: 16.04 ± 0.69 mm, WT: 9.32 ± 0.87 mm, average increase: 172%, *P* = 0.0003; Figure 5, A and B, and Supplemental Figure 9) with increased vascular density (*Nrp1^SM22KO^*: 34.92 ± 1.68, WT: 27.73 ± 1.476, average increase: 126%, *P* = 0.0012; Figure 5, A and C, and Supplemental Figure 9). In addition, there was a significant decrease in the pericyte lining (NG2^+^ cells) of blood vessels in the retinas from *Nrp1^SM22KO^* compared with WT sex-matched littermate (*Nrp1^SM22KO^*: 4.05 ± 0.53, WT: 9.94 ± 0.91, average decrease: 59%, *P* = 0.016; Figure 5, A and D, and Supplemental Figure 9).

### NRP-1 expression is induced in the inflow artery of AVF of WT but not Nrp1^SM22KO^ mice.

**Figure 6:**
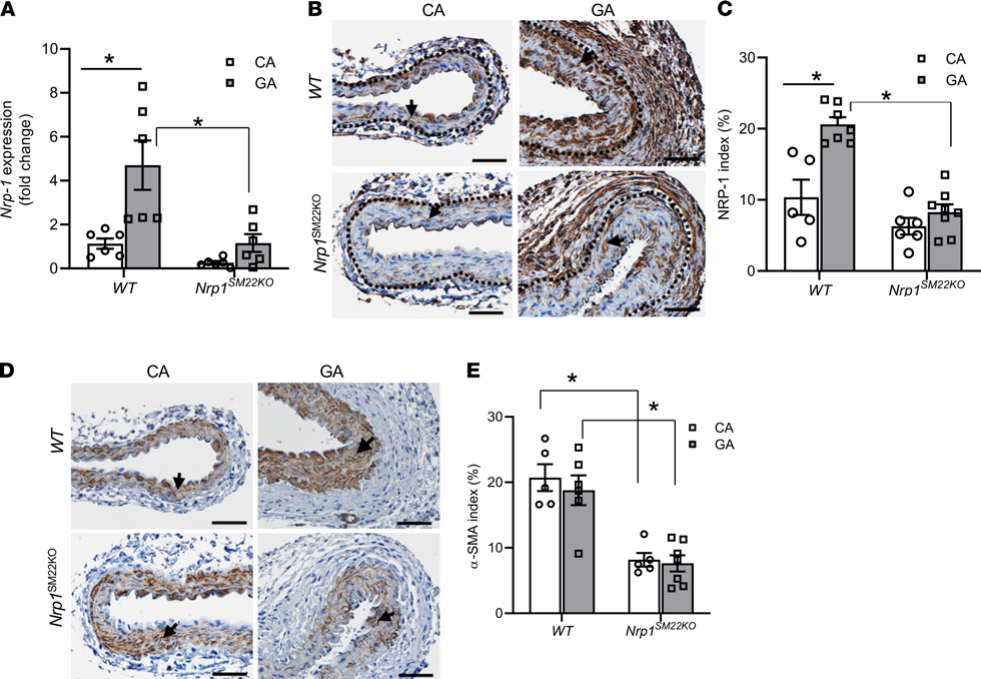
Flow-induced *Nrp1* upregulation in the AVF in the inflow arteries. (**A**) NRP-1 gene expression was assessed by qPCR in the AVF inflow arteries (GA) and contralateral carotid arteries (CA) at 3 days after AVF creation. There is a significant increase in NRP-1 gene expression in GA compared with CA from WT mice. However, there was no significant difference in NRP-1 expression in GA compared with CA in *Nrp1^SM22KO^* mice. Immunostaining was performed to assess NRP-1 and α-SMA levels in GA and CA at 14 days after arteriovenous fistula (AVF) creation. (**B** and **D**) Representative images of NRP-1 (**B**) and α-SMA (**D**) in GA and CA from *Nrp1^fl/fl^* (WT) and *Nrp1^fl/fl^/SM22α^Cre+^* (*Nrp1^SM22KO^*) mice. The dotted line in **B** indicates the media and adventitia layers in the vessel wall. All images were captured using 10× magnification. Scale bar: 50 μm. The arrows point to brown cells positive for NRP-1 (**B**) and α-SMA (**D**) staining in the vessel wall. (**B**, **C**, and **E**) The intensity of brown stain positive NRP-1 (**C**) index in α-SMA (**B** and **E**) index in **C** were quantified as described and presented as mean ± SEM of *n* = 5–7 animals. A 2-way ANOVA with multiple comparison was performed. *P < 0.05.

Surgical AVFs were created to connect the carotid artery to the ipsilateral jugular vein. At 3 days after AVF creation, there was a significant increase in the average gene expression of *Nrp1* in the inflow artery of the AVF (graft artery [GA]) compared with the contralateral artery (control artery [CA]) in WT mouse (GA: 4.7 ± 1.13, CA: 1.13 ± 0.23, average increase: 416%, *P* = 0.0026; Figure 6A). However, in *Nrp1^SM22KO^* mice, there was no difference in the average gene expression of *Nrp1* in GA compared with CA, suggesting that *Nrp1* in SMCs contributed to the increased *Nrp1* gene expression GA. We next performed immunostaining to determine the NRP-1 protein levels in the vessel wall. In WT mice, there was a significant increase in cells staining positive for NRP-1 in GA compared with CA (WT-GA: 20.61 ± 1.01, WT-CA: 10.36 ± 2.47, average increase: 199%, *P* = 0.0001; Figure 6, B and C). Cells staining positive for NRP-1 were located in the media and intima where there are α-SMA^+^ cells (Figure 6D). There was a significant decrease in the NRP-1 staining in the media and intima regions of the GA of *Nrp1^SM22KO^* mice compared with WT (*Nrp1^SM22KO^*: 8.27 ± 1.09, WT: 20.61 ± 1.01, average decrease: 60%, *P* < 0.0001; Figure 6, B and C) and CA (*Nrp1^SM22KO^*: 6.28 ± 1.17, WT: 10.36 ± 2.47, average decrease: 39%, *P* = 0.069; Figure 6, B and C). However, there was no difference in the NRP-1 staining in the adventitia of either the GA or CA (Figure 6B and data not shown). Interestingly, α-SMA staining was also significantly decreased in the media and intima regions of the GA from *Nrp1^SM22KO^* mice compared with WT sex-matched littermate (*Nrp1^SM22KO^*: 7.63 ± 1.23, WT: 18.8 ± 2.27, average decrease: 59%, *P* = 0.007; Figure 6, D and E) and in CA (*Nrp1^SM22KO^*: 8.18 ± 1.03, WT: 20.71 ± 2.06, average decrease 64%, *P* = 0.0007; Figure 6, D and E).

### Nrp1^SM22KO^ mice have decreased gene expression of Eng, Alk1, and Tgf-β1 with increased SMAD8/9 in the inflow artery of AVF.

**Figure 7:**
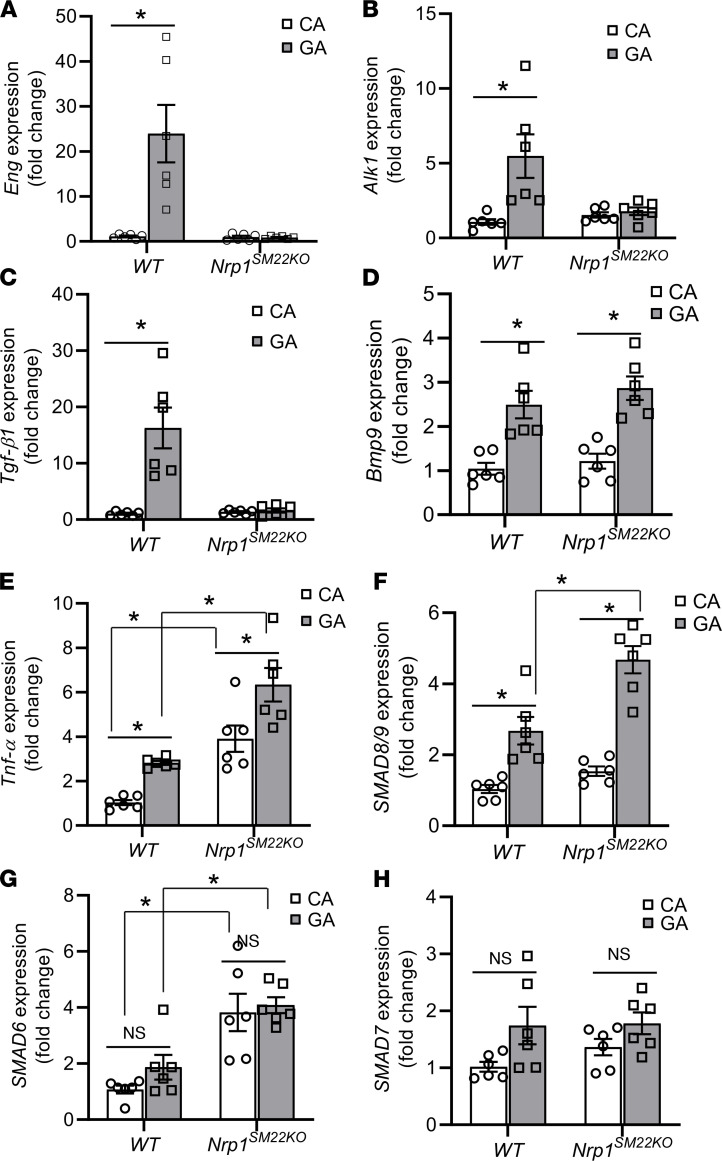
*Nrp1* deletion in smooth muscle cell reduces gene expression of *Eng*, *Alk1*, and *Tgfb1* but not *Tnfa* in the AVF inflow arteries. Gene expression was assessed by qPCR in AVF inflow artery (GA) and contralateral carotid artery (CA) at 3 days after arteriovenous fistula (AVF) creation. (**A**–**C**) There was a significant increase in *Eng* (**A**), *Alk1* (**B**), and *Tgf-β1* (**C**) expression in GA compared with CA from *Nrp1^fl/fl^* (WT) mice but not in *Nrp1^fl/fl^/SM22α^Cre+^* (*Nrp1^SM22KO^*) mice. (**D**–**F**) There was a significant increase in *Bmp9* (**D**), *Tnfa* (**E**), and *SMAD8/9* (**F**) expression in GA compared with CA in both WT and *Nrp1^SM22KO^* mice. (**G** and **H**) However, there was no significant difference in (**G**) *SMAD6* and (**H**) *SMAD7* in CA compared with GA, regardless of *Nrp1* deletion. The data were normalized to the gene expression in the CA of WT mouse and expressed as mean fold change ± SEM in GA and CA from WT and *Nrp1^SM22KO^* of *n* = 6 animals. Two-way ANOVA was performed. *P < 0.05

We examined the gene expression of *Eng* and *Alk1* using quantitative PCR (qPCR) at 3 days after AVF placement in the inflow artery (GA) compared with CA. At 3 days after AVF creation, in WT mice, there was a significant increase in the gene expression of *Eng* in GA compared with CA (GA: 23.94 ± 6.39, CA: 1.10 ± 0.19, average increase: 2176%, *P* = 0.0003; Figure 7A) and *Alk1* (GA: 5.48 ± 1.46; CA: 1.08 ± 0.19, average increase: 507%, *P* = 0.0026; Figure 7B). However, in *Nrp1^SM22KO^* mice, there was no difference in the gene expression of *Eng* and *Alk1* in the GA compared with the CA. TGF-β1 and BMP9 are known ligands of ALK1/ENG and ALK5 (TGF-βR1) signaling, and their levels were assessed in both WT and *Nrp1^SM22KO^* mice. In WT animals, there was a significant increase of *Tgfb1* gene expression in GA compared with the CA (GA: 16.27 ± 3.61, CA: 1.05 ± 0.14, average increase: 1549%, *P* < 0.0001; Figure 7C) and *Bmp9* (GA: 2.49 ± 0.31, CA: 1.04 ± 0.14, average increase: 239%, *P* = 0.0014; Figure 7D). In *Nrp1^SM22KO^* mice, there was no difference in the average *Tgfb1* (Figure 7C) gene expression, but there was a significant increase in *Bmp9* gene expression in the GA compared with CA (GA: 2.87 ± 0.27, CA: 1.21 ± 0.17, average increase: 237%, *P* = 0.0003; Figure 7D). There was an increase in the gene expression of *Tnfa* in the GA compared with the CA of both WT animals (GA: 2.82 ± 0.09, CA: 1.03 ± 0.12, average increase: 273%, *P* < 0.0001) and *Nrp1^SM22KO^* (GA: 6.34 ± 0.75, CA: 3.91 ± 0.60, average increase: 162%, *P* = 0.011; Figure 7E), although *Nrp1^SM22KO^* mice exhibited greater levels of *Tnfa* in both GA and CA.

We next assessed gene expression of vascular SMAD8/9, SMAD6, and SMAD7. There was a significant increase in SMAD8/9 expression in GA compared with CA in both WT (GA: 2.66 ± 0.39, CA: 1.03 ± 0.11, average increase: 258%, *P* = 0.0032) and in *Nrp1^SM22KO^* animals (GA: 4.68 ± 0.38, CA: 1.54 ± 0.13, average increase: 303.8%, *P* < 0.0001; Figure 7F). However, *Nrp1^SM22KO^* mice also showed a significant increase in SMAD8/9 expression in the GA as compared with WT mice *(Nrp1^SM22KO^*: 4.68 ± 0.29, WT: 2.66 ± 0.39, average increase: 153%, *P* = 0.0005; Figure 7F). There was no significant difference in the gene expression of SMAD6 and SMAD7 in the GA compared with the CA in both WT and *Nrp1^SM22KO^* mice (Figure 7, G and H). SMAD6 expression was significantly higher in both the GAs from *Nrp1^SM22KO^* mice as compared with WT animals (*Nrp1^SM22KO^*: 4.08 ± 0.29, WT: 1.87 ± 0.44, average increase: 218%, *P* = 0.008; Figure 7G) and CA (*Nrp1^SM22KO^*: 3.82 ± 0.667, WT: 1.07 ± 0.14, average increase: 357%, *P* = 0.0011; Figure 7G).

### Nrp1^SM22KO^ mice have decreased immunostaining of ENG, ALK1, and TGF-β1 in the inflow artery of AVF.

**Figure 8:**
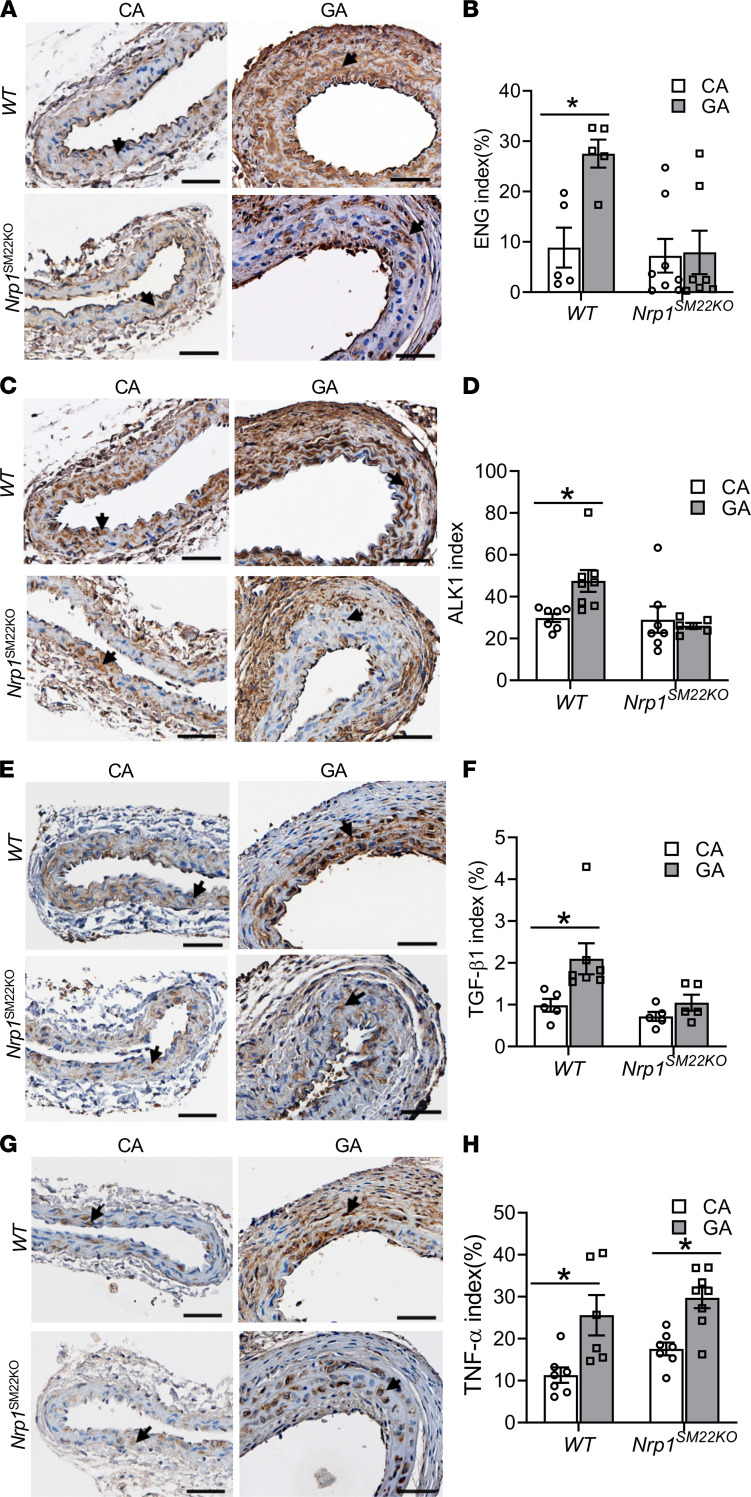
Smooth muscle cell *Nrp1* deletion attenuates flow induced ENG, ALK1, and TGF-β1 but not TNF-α levels in AVF inflow arteries. Immunostaining was performed to assess ENG, ALK1, TGF-β1, and TNF-α levels in the AVF inflow arteries (GA) and contralateral carotid arteries (CA) at 14 days after arteriovenous fistula (AVF) creation. (**A**, **C**, **E**, and **G**) Representative images of (**A**) ENG, (**C**) ALK1, (**E**) TGF-β1, and (**G**) TNF-α in GA and CA from *NRP1^fl/fl^* (WT) and *Nrp1^fl/fl^/SM22α^Cre+^* (*Nrp1^SM22KO^*) mice. All images were captured using 10× magnification. Scale bar: 50 μm. The arrows show the brown positive stain of ENG (**A**), ALK1 (**C**), TGF-β1 (**E**), and TNF-α (**G**). (**B**, **D**, **F**, and **H**) The intensity of brown stain positive for ENG (**B**), ALK1 (**D**), TGF-β1 (**F**), and TNF-α (**H**) index were quantified as described and presented as mean ± SEM of *n* = 5–7 animals. Two-way ANOVA was performed. *P < 0.05.

We further performed immunostaining to determine the protein levels of ENG, ALK1, TGF-β1, and TNF-α in the GA and CA from WT and *Nrp1^SM22KO^* mice. Fourteen days after AVF placement in WT mice, IHC analysis revealed that there was a significant increase in the ENG^+^ cells (GA: 27.53 ± 2.79, CA: 8.86 ± 3.96, average increase: 311%, *P* = 0.0052; Figure 8, A and B) and ALK1 staining (GA: 47.53 ± 5.22; CA: 29.82 ± 1.94, average increase: 159%, *P* = 0.0084; Figure 8, C and D) in GA compared with CA. In *Nrp1^SM22KO^* mice, there was no significant difference in ENG (Figure 8, A and B) or ALK1 staining (Figure 8, C and D) in GA compared with CAs. In WT animals, the average TGF-β1 staining was significantly increased in the GA compared with CA (GA: 2.1 ± 0.37; CA: 0.98 ± 0.15, average increase: 213%, *P* = 0.0078; Figure 8, E and F), but in *Nrp1^SM22KO^* mice, there was no significant difference between GA and CA (Figure 8, E and F). There was a significant increase in TNF-α staining in the GA compared with the CA in WT animals (GA: 25.59 ± 4.81, CA: 11.32 ± 1.83, average increase: 226%, *P* = 0.0017) and in *Nrp1^SM22KO^* mice (GA: 29.73 ± 2.46, CA: 17.54 ± 1.52, average increase: 169%, *P* = 0.0036; Figure 8, G and H). However, there was no significant difference in the TNF-α staining in the GA from *Nrp1^SM22KO^* mice compared with GA from WT animals (Figure 8, G and H). AVF creation significantly increased CD45 index in GA compared with CA in both WT mice (GA: 6.38 ± 1.22, CA: 1.72 ± 0.37, average increase: 370.9%, *P* = 0.034; Supplemental Figure 10, A and B) and in *Nrp1^SM22KO^* mice (GA: 6.76 ± 1.57, CA: 1.96 ± 0.45, average increase: 344.89%, *P* = 0.028; Supplemental Figure 10, A and B). However, there was no significant difference in CD45 staining in GA between *Nrp1^SM22KO^* and WT sex-matched littermate animals (Supplemental Figure 10, A and B). Furthermore, there was no significant difference in CD68 staining index between GA and CA between *Nrp1^SM22KO^* and WT sex-matched littermate mice (Supplemental Figure 10, C and D).

### NRP-1 enhances ENG/ALK1 signaling in SMCs from the aorta of Nrp1^SM22KO^ compared with WT sex-matched littermate mice.

**Figure 9:**
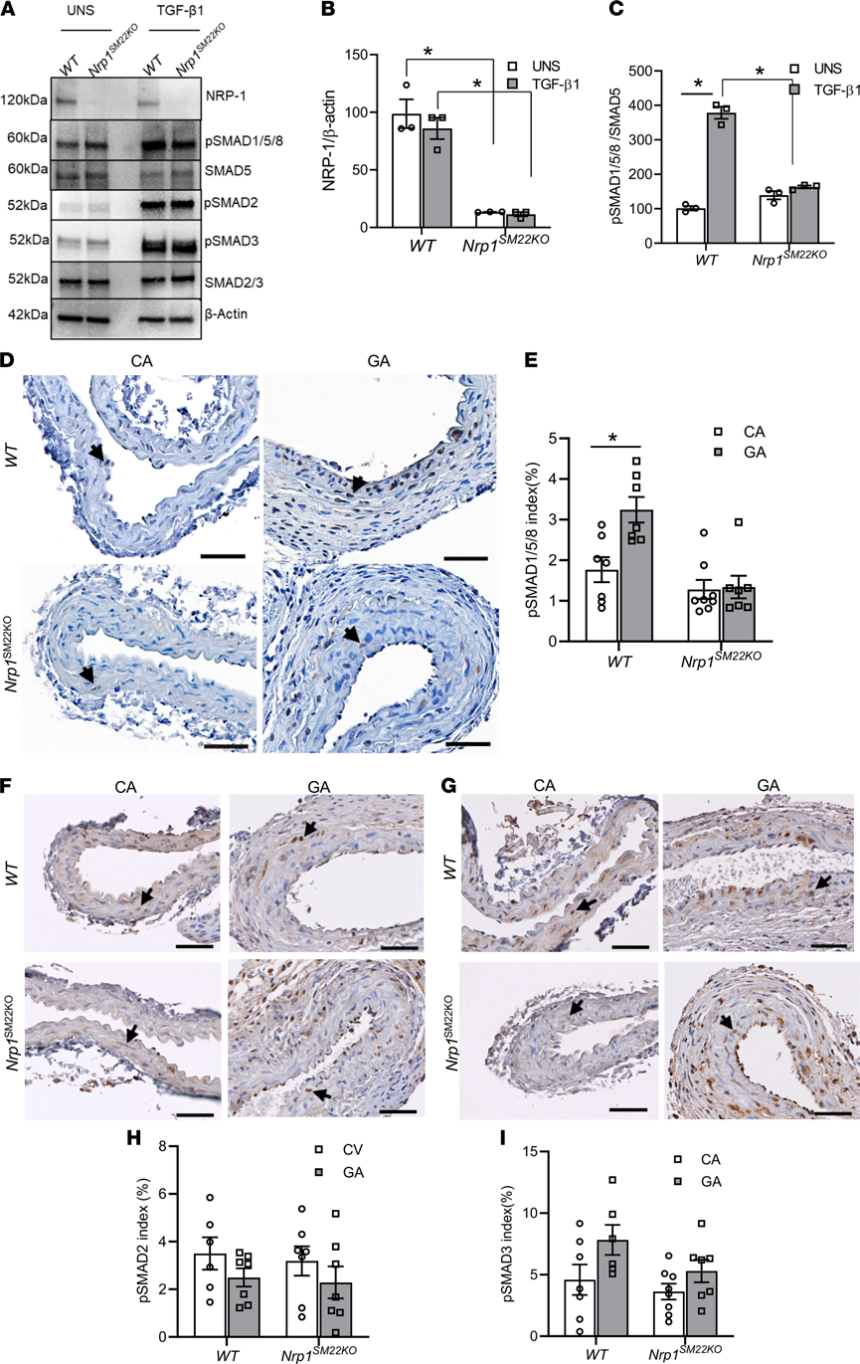
*Nrp1* deletion in smooth muscle cells results in a reduction of TGF-β1–pSMAD1/5/8 signaling. (**A**) Aortic smooth muscle cells were isolated from *Nrp1^fl/fl^* (WT) or *Nrp1^fl/fl^/SM22α^Cre+^* (*Nrp1^SM22KO^*) mice. After overnight serum starvation, cells were stimulated with 10 ng/mL TGF-β1 for 30 minutes, and pSMAD1/5/8 levels were assessed by Western blot. Representative Western blot shows TGF-β1 stimulation (TGF-β1) increased pSMAD1/5/8 compared with unstimulated control (UNS) cells from WT mice but not in cells from *Nrp1^SM22KO^* mice with no change in pSMADs 2 and 3. (**B** and **C**) Densitometric analysis of NRP-1 (**B**) and pSMAD1/5/8 (**C**) were performed using NIH ImageJ software. Data are shown as mean ± SEM of *n* = 3. (**D**–**G**) Immunostaining analysis of AVF inflow arteries (GA) and contralateral control arteries (CA) at 14 days after AVF creation. All images were captured using 10× magnification. Scale bar: 50 μm. Arrows indicate the brown stained nuclei of pSMAD1/5/8 (**D**), pSMAD2 (**F**), and pSMAD3 (**G**). The intensity of brown stain pSMAD1/5/8 (**E**), pSMAD2 (**H**), and pSMAD3 (**I**) was measured as described and presented as mean ± SEM of *n* = 5–8 animals. Two-way ANOVA was performed. *P < 0.05.

Western blot analysis of NRP-1 demonstrated that it was undetected in SMCs isolated from aorta of *Nrp1^SM22KO^* compared with WT sex-matched littermate mice (*Nrp1^SM22KO^*: 13.47 ± 0.13, WT: 98.74 ± 12.5, average decrease: 86%, *P* < 0.0001; Figure 9, A and B). Exogenous TGF-β1 stimulation in SMCs isolated from the aorta of *Nrp1^SM22KO^* compared with WT sex-matched littermate mice did not change the expression of NRP-1 (Figure 9, A and B). We next examined pSMAD1/5/8, which is downstream to the TGF-β1/ENG and TGF-β1/ALK1 signaling pathway. TGF-β1 stimulation significantly increased pSMAD1/5/8 levels in SMCs from WT mice (TGF-β1: 378.51 ± 17.65 vs. UNS: 101.61 ± 6.30, average increase: 373%, *P* = 0.00012; Figure 9, A and C). However, pSMAD1/5/8 levels did not significantly change in TGF-β1 stimulated SMCs from *Nrp1^SM22KO^* mice compared with unstimulated SMCs (Figure 9, A and C). Notably, NRP-1 deletion had no influence on TGF-β1–mediated phosphorylation of SMAD2 or SMAD3 in SMCs (Figure 9A).

Next, we assessed pSMAD1/5/8 levels in GAs of both WT and *Nrp1^SM22KO^* mice by immunostaining. Fourteen days after the placement of the AVF, WT mice showed a significant increase in pSMAD1/5/8 levels in GA as compared with the CA (GA: 3.24 ± 0.32, CA: 1.77 ± 0.31, average increase: 183%, *P* = 0.0013; Figure 9, D and E). However, in *Nrp1^SM22KO^* mice, there was no change seen in the pSMAD1/5/8 levels in GA compared with CA (Figure 9, D and E). Interestingly, pSMAD1/5/8 was localized to the intima and media of the GA in *Nrp1^SM22KO^* mice, and pSMAD1/5/8 staining was significantly lower in *Nrp1^SM22KO^* mice compared with WT animals (*Nrp1^SM22KO^*: 1.34 ± 0.28, WT: 3.24 ± 0.32, average decrease 59%, *P* < 0.0001; Figure 9, D and E). We next assessed for pSMAD2 by immunostaining. There was no significant difference between pSMAD2 (Figure 9, F and H) and pSMAD3 (Figure 9, G and I) immunostaining in the GA compared with CA in both WT and *Nrp1^SM22KO^* mice. These results support the notion that ablation of NRP-1 in SMCs reduces pSMAD1/5/8 in SMCs and AVF. We further tested the expression levels of vascular SMADs, *SMAD6*, *SMAD7*, and *Bmp9* in SMCs by qPCR. We found no differences between the expression levels of *SMAD6*, *SMAD7*, and *Bmp9* before and after TGF-β1 stimulation in mouse aorta SMCs from *Nrp1^SM22KO^* and WT sex-matched littermate mice (Supplemental Figure 11).

### Nrp1 deletion in SMCs have increased apoptosis.

**Figure 10:**
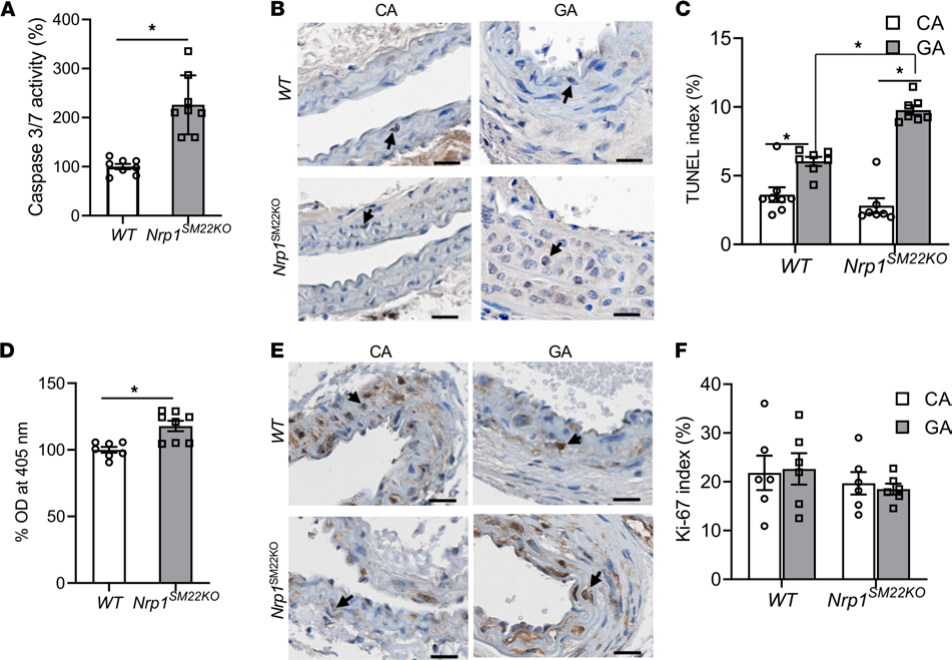
Smooth muscle cell *Nrp1* deletion causes an increase in apoptosis of smooth muscle cells. Aortic smooth muscle cells (SMCs) isolated from *Nrp1^fl/fl^* (WT) or *Nrp1^fl/fl^/SM22α^Cre+^* (*Nrp1^SM22KO^*) mice. (**A**) Caspase-3/7 activity was assessed in SMCs as described to assess apoptosis of SMCs in vitro. (**B**) Representative images of TUNEL staining on tissue sections of the inflow artery (GA) and contralateral carotid artery (CA) at 14 days after AVF creation. All images were captured using 10× magnification. Scale bar: 20 μm. Arrows show brown staining TUNEL^+^ cells. (**C**) The intensity of the brown TUNEL stain was measured as described. After overnight serum starvation, SMCs were stimulated with 10 ng/mL TGF-β1 for 24 hours. (**D**) Proliferation was assessed in vitro as described. There was 18% increase in proliferation (*P* = 0.01) of SMCs isolated from *Nrp1^SM22KO^* mice compared with cells isolated from WT sex-matched littermates. (**E**) Representative images of Ki-67 staining on tissue sections of GA from AVF and CA at 14 days after AVF creation. All images were captured using 10× magnification. Scale bar: 20 μm. Arrows show brown staining of KI-67^+^ cells. (**F**) The intensity of the brown Ki-67 stain was measured as described. Data are shown as mean ± SEM of *n* = 8 in vitro experiments or *n* = 6 animals. Nonparametric Mann-Whitney *U* test (**A** and **D**) or 2-way ANOVA (**C** and **F**) was performed. *P < 0.05.

We determined the caspase-3/7 activity to assess for apoptosis. There was a significant increase in the mean caspase-3/7 activity in SMCs isolated from *Nrp1^SM22KO^* compared with WT sex-matched littermate mice (*Nrp1^SM22KO^*: 226.01± 21.24, WT: 100 ± 5.53, average increase: 226%, *P* < 0.0002; Figure 10A). We next performed TUNEL staining to assess for apoptosis in the arteries from AVF. There was a significant increase in the number of TUNEL^+^ cells in GA compared with CA of both *Nrp1^SM22KO^* (GA: 9.77 ± 0.34, CA: 2.82 ± 0.54, average increase: 446%, *P* < 0.0001; Figure 10, B and C) and WT sex-matched littermate mice (GA: 6.04 ± 0.33, CA: 3.62 ± 0.54, average increase: 267%, *P* = 0.0009; Figure 10, B and C). TUNEL^+^ cells in the GA from *Nrp1^SM22KO^* mice were significantly greater than WT sex-matched littermate (*Nrp1^SM22KO^*: 9.77 ± 0. 34, WT: 6.04 ± 0.33, average increase: 253%, *P* < 0.0001; Figure 10, B and C). These results suggest that SMCs deficient of NRP-1 are more susceptible to apoptosis. Finally, we assessed proliferation. There was 18% increase in proliferation (*P* = 0.009) of SMCs isolated from *Nrp1^SM22KO^* mice compared with cells isolated from WT sex-matched littermates (Figure 10D). However, there was no significant difference in Ki-67 staining in the GA compared with the CA from WT sex-matched littermates or *Nrp1^SM22KO^* mice (Figure 10, E and F).

### NRP-1 is part of ALK1/ENG signaling complex in SMCs.

**Figure 11:**
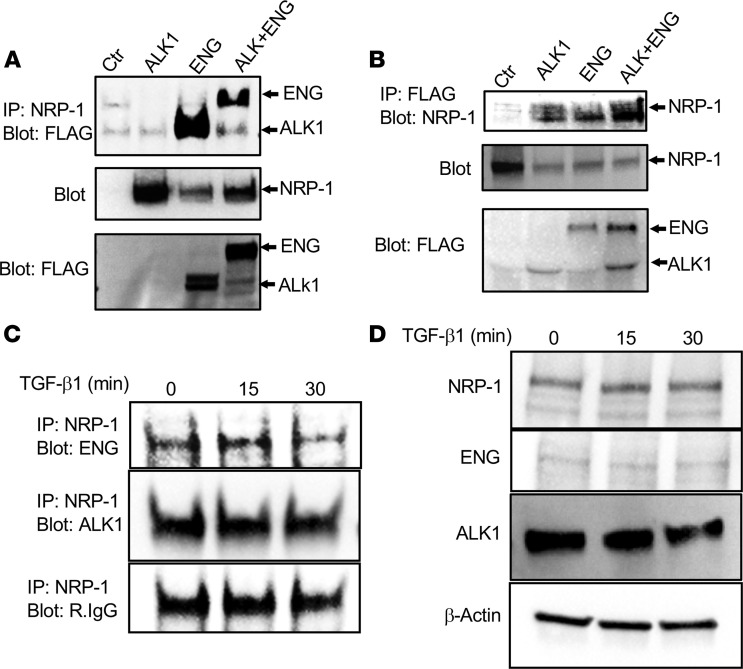
Biochemical association of NRP-1 with ALK1 and ENG. HEK293T cells (Ctr) were transfected with NRP-1 and FLAG-tagged constructs of ALK1 (ALK1), Endoglin (ENG), and ALK1 + ENG. (**A** and **B**) Cell lysates were subjected to immunoprecipitation with anti**–**NRP-1 antibody (IP: NRP-1) and probed with anti-FLAG Western blot (Blot: FLAG) (**A**) and FLAG (IP:FLAG) and probed with anti–NRP-1 (Blot: NRP-1) Western blot (**B**). Both ENG and ALK1 were detected in NRP-1 immunoprecipitate (**A**) and NRP-1 detected in immunoprecipitates of both FLAG-ALK1 and FLAG-ENG (**B**). (**C**) ALK1 and ENG were also detected in NRP-1 immunoprecipitate from cell lysates of TGF-β1–stimulated aortic SMCs. TGF-β1 stimulation to the SMCs did not affect the biochemical association of NRP-1 with ALK1 or ENG. (**D**) Total protein levels of NRP-1, ALK1, and ENG in SMCs not changed with TGF-β1 stimulation. All experiments were repeated 3 times, and representative immunoblots are shown.

We found that NRP-1 coimmunoprecipitated with ENG and ALK1 in the HEK-293T cell overexpression system (Figure 11A). To confirm these results, we repeated the experiment using the HEK-293T cell overexpression system, and we isolated cell lysates that were immunoprecipitated with the FLAG antibody and probed for NRP-1 to assess for the interaction of ALK1 or ENG with NRP-1. As expected, FLAG tagged to ALK1 or ENG was able to pull down NRP-1 in the immune complex (Figure 11B). To assess the endogenous interaction of NRP-1 with ALK1 or ENG in SMCs, coimmunoprecipitation experiments were performed in aortic SMCs, and we found that ALK1 and ENG coimmunoprecipitated with NRP-1 (lane 1, Figure 11C). We next investigated whether TGF-β1 stimulation had an impact on the biochemical association of NRP-1 with ENG or ALK1. We stimulated SMCs with TGF-β1 for 15 and 30 minutes. There was no change in either ENG or ALK1 levels associated with NRP-1 after TGF-β1 stimulation at 15 minutes (lane 2, Figure 11C) or 30 minutes (lane 3, Figure 11C). There was no change in the total protein (Figure 11D) at either 15 or 30 minutes compared with unstimulated (0 minutes, lane 1, Figure 11D) cells. To understand how NRP-1 deletion could affect ENG/ALK1 signaling, we tested whether there was a change in ENG and ALK1 interactions in flow-induced AVF arteries and in SMCs stimulated with TGF-β1. There was a significant increase in ENG and ALK1 colocalization in GA compared with CA in WT animals (GA: 60.03 ± 2.64, CA: 36.59 ± 1.39, average increase 264%, *P* = 0.0010; Supplemental Figure 12, A and C) and in *Nrp1^SM22KO^* animals (GA: 42.92 ± 3.94, CA: 19.52 ± 4.84, average increase 320%, *P* = 0.0010; Supplemental Figure 12, B and C). There was a significant reduction of ENG and ALK1 colocalization in GA of *Nrp1^SM22KO^* compared with WT sex-matched littermate mice (*Nrp1^SM22KO^*: 42.92 ± 3.94, WT: 60.03 ± 2.64, average decrease: 28%, *P* = 0.014; Supplemental Figure 12C). Coimmunoprecipitation experiments in SMCs demonstrated minimal interaction between ENG and ALK1 in SMCs from *Nrp1^SM22KO^* mice compared with WT sex-matched littermate mice (Supplemental Figure 12D).

## Discussion

In the present study, we demonstrated that there was a reduction of NRP-1 in perivascular SMCs of HHT patient liver sections compared with controls. *Nrp1^SM22KO^* mice have shown a direct connection of artery to vein that resembles a large AVF commonly seen in cerebral and pulmonary AVMs in patients with HHT (5, 30); these mice also show dilated vessels with decreased SMC lining, along with increased infiltration of CD45^+^ and CD68^+^ cells in adult lung and liver. *Nrp1^SM22KO^* mice at P5 retina have dilated capillaries with a significant reduction of NG2^+^ pericytes lining. There is a significant increase in gene expression of *Nrp1*, *Tgfb1*, *Eng*, *Alk1*, and *Tnfa* in association with increased pSMAD1/5/8 in the AVF inflow artery (GA) compared with contralateral carotid artery (CA) in *Nrp1^fl/fl^* (WT) mice but not in the *Nrp1^SM22KO^* mice. Moreover, SMCs from the aorta of *Nrp1^SM22KO^* mice have a significant reduction of TGF-β1–induced pSMAD1/5/8 and increased apoptosis compared with SMCs from WT mice. Coimmunoprecipitation experiments reveal that NRP-1 interacts with ALK1 and ENG in HEK293T cell overexpression and in primary SMCs isolated from adult mouse aorta. In addition, there is a reduction of ALK1/ENG interaction in SMCs in the absence of NRP-1. These results suggest that NRP-1 deletion in SMC leads to reduced ALK1/ENG signaling with increased cell death leading to AVM formation. *Nrp1^fl/fl^* mice were bred with SM22α Cre (*SM22α^Cre^*) mice to generate the SMC-specific NRP-1–KO mice (28).

There is debate on whether the SM22α promoter expression can occur at low levels in other cell types during embryonic development, and it raises the possibility of *Nrp1* gene deletion using SM22α Cre being nonspecific (33). To address this, we performed coimmunofluorescence staining for SM22α and CD31 in adult mouse lungs, and the results indicate that there is a minimal colocalization of SM22α and CD31 in SMCs and ECs (Supplemental Figure 2). Importantly, adjacent sections costained for NRP-1 and SM22α demonstrated that there is no change in NRP-1 expression in CD31^+^ cells in Nrp*1^SM22KO^* compared with WT mice (Supplemental Figures 2 and 3). Furthermore, we also show the absence of NRP-1 in aortic SMCs with no change in pulmonary ECs (27) isolated from the *Nrp1^SM22KO^* mice compared with cells from WT animals. Moreover, SM22α was minimally detected in pulmonary ECs, possibly due to a small amount of SMC contamination in ECs (Supplemental Figure 4). These results suggest that NRP-1 ablation is specific to SMCs in adult *Nrp1^SM22KO^* mice.

NRP-1 is predominantly expressed by ECs, neuronal cells, epithelial cells, tissue macrophages, fibroblasts, and SMCs (29). In ECs, NRP-1 functions as a coreceptor for several receptor tyrosine kinases that are involved in vasculogenesis and angiogenesis, including VEGFR2 (34). It was presumed that the interaction between VEGFR2 and NRP-1 can occur within the same cell (*cis*) or through cell-to-cell (*trans*) contact (35). In support of this notion, it was demonstrated that the ectopic addition of soluble NRP-1 dimer could rescue aberrant vascular development in *Nrp1*-deleted paraaortic splanchnopleuric mesoderm (36). However, ablation of *Nrp1* in mice causes embryonic lethality at E11.5 due to vascular defects, and conditional deletion of *Nrp1* in ECs also causes death immediately after birth (37, 38). Studies in mice with an inducible Cre/Lox gene deletion model suggest that loss of *Nrp1* in ECs (27) impairs vascular sprouting with a reduction in tip cells in mouse retinal vasculature (39). These results suggest that EC NRP-1 is required for developmental angiogenesis and vasculogenesis.

Moreover, recent studies have shown that NRP-1 inhibits ALK1- and ALK5-mediated SMAD2/3 phosphorylation in ECs and modulates tip and stalk cell phenotypes in vascular sprouting and stretch-induced TGF-β1/ALK1 signaling in ECs when cocultured with SMCs (23, 40). On the other hand, flow dynamics and acute injury are known to upregulate ALK1 expression in SMCs and to increase proliferation and migration of SMCs via TGF-β1 signaling (41). However, the role of NRP-1 in ALK1 signaling in SMCs and pericytes in vessel maturation and remodeling is unclear. Moreover, genetic mutations in *ALK1* and *ENG* result in HHT pathogenesis with decreased perivascular SMCs (8–10). These studies prompted us to assess NRP-1 protein levels in HHT samples. We found that there was a reduction of NRP-1 in perivascular SMCs in liver tissue sections of patients with HHT with ALK1 mutation. Further studies are needed to determine the role of NRP-1 levels in patients with HHT to determine the molecular mechanisms that regulate NRP-1 in HHT pathogenesis.

Previous studies in adult *Nrp1^SM22KO^* mice have shown that there is perivascular inflammatory cell infiltration in the lung and that the mice are prone to heart failure after myocardial infarction with metabolic defects (28). Moreover, studies in mice with inducible KO of *Nrp1* (*Nrp1iKO*) in SMCs show impaired postnatal lung development with a decrease in SMCs (42). Consistent with these observations, we found that there is a presence of hemorrhagic cells in the tissue interstitial space, along with increased CD68^+^ and CD45^+^ cells in the lung and liver with a decrease in the α-SMA^+^ cells in the adult lung from *Nrp1^SM22KO^* mice compared with WT sex-matched littermates. In addition to the reduction of α-SMA^+^ cells, there was an increased average vessel cross-sectional area, with a reduction of NG2^+^ pericyte content in the adult lungs of *Nrp1^SM22KO^* mice compared with WT sex-matched littermates. Moreover, we observed AVF in the lung of *Nrp1^SM22KO^* mice. We did observe an increase in average vessel cross-sectional area in the liver but did not see a direct artery-to-vein connection, which may be due to the differences in vascular architecture in the lung and liver. Consistent with these results, we also observed that there is a dilated capillary network with decreased pericyte lining the retina vasculature at P5. These results suggest that *Nrp1^SM22KO^* mice recapitulate the histological finding of AVMs in patients with HHT similar to mouse models of *Alk1^+/–^* and *Eng^+/–^* (18, 19).

AVF results in increased blood flow and reduced SMCs, leading to friable and dilated vessels, and it is a typical feature of many AVMs, resulting in bleeding and stroke (5, 11). To further investigate the role of NRP-1 reduction in SMCs in AVFs, we created a surgical AVF, and in the inflow artery (GA) compared with contralateral CA, we observed an increase in the gene expression of NRP-1, ENG, ALK1, and TGF-β1. However, we found no change in ENG, ALK1, and TGF-β1 expression in GA from *Nrp1^SM22KO^* mice compared WT sex-matched littermate mice, perhaps due to a lack of NRP-1 in SMCs. It has been demonstrated that AVMs have increased TNF-α (43), and TNF-α suppresses ENG expression in AVMs (44). In the present study, there was a significant increase in *Tnfa* expression at 3 days after AVF creation in GA compared with CA in both the WT and *Nrp1^SM22KO^* mice, but there was no significant difference in TNF-α protein expression by immunostaining in GA from *Nrp1^SM22KO^* compared with WT animals, despite a reduction in ENG expression. These results indicate that the induction of ENG, ALK1, or TGF-β1 expression in GA is due to increased blood flow. We next assessed immune cell infiltration in the vessel wall of GA compared with CA at 14 days after AVF creation and observed that there was a significant increase in CD45^+^ leukocyte infiltration, consistent with previous studies (45). There was no significant difference in CD68 staining in GA compared with CA in WT sex-matched littermate or *Nrp1^SM22KO^* mice (Supplemental Figure 11). However, like the TNF-α index, there was no difference in the CD45 index in GA from *Nrp1^SM22KO^* animals compared with WT sex-matched littermate animals.

In vitro cell culture and in vivo studies have shown that shear stress results in increased *SMAD6* expression in ECs and *SMAD7* expression in adventitial fibroblasts, and these SMADs are involved in maintaining vascular integrity (46–48). In the present study, 3 days after AVF creation, there was no difference in the gene expression of *SMAD6* and *SMAD7* in the inflow artery compared with control artery of either WT or *Nrp1^SM22KO^* mice. However, there was a significant increase in the basal *SMAD6* gene expression in inflow artery compared and control artery from *Nrp1^SM22KO^* mice compared with WT animals. There was a significant upregulation of SMAD6 in isolated SMCs from *Nrp1^SM22KO^* mice compared with WT animals.

ENG and ALK1 are cell-surface transmembrane receptors involved in TGF-β1 and BMP signaling pathways that can activate pSMAD1/5/8, and impairment or mutations of *ENG* and or *ALK1* signaling can lead to vascular malformations associated with HHT disease (21). In the present study, we observed a significant increase in the *Bmp9* gene expression in the inflow arteries of both WT and *Nrp1^SM22KO^* mice, yet the decrease of pSMAD1/5/8 levels by immunostaining in inflow artery of *Nrp1^SM22KO^* mice could be attributed to decreased TGF-β1, ENG, and ALK1 expression. Moreover, there was increased pSMAD1/5/8 in inflow arteries from WT animals localized to the media where SMCs are prominent. Previous studies have shown that dorsomorphin, a BMP receptor inhibitor, was able to block SMAD1/5/8 phosphorylation in SMCs when stimulated with BMP9 but not in SMCs stimulated with TGF-β1 (49), indicating that TGF-β1–mediated SMAD1/5/8 phosphorylation is independent of BMP receptor activation. These results prompted us to investigate NRP-1 contribution in TGF-β1/ENG and TGF-β1/ALK1 signaling in isolated SMCs. Aortic SMCs from *Nrp1^SM22KO^* mice had decreased pSMAD1/5/8 levels compared with cells isolated from littermate WT mice after TGF-β1 stimulation. Furthermore, coimmunoprecipitation studies performed in HEK293T cells revealed that NRP-1 had a direct interaction with ENG and ALK1. We next sought to investigate the effect of receptor stimulation on biological interaction of ENG or ALK1 with NRP-1 using coimmunoprecipitation studies in mouse aortic SMCs after TGF-β1 stimulation. However, we observed that there was no change in ENG or ALK1 levels associated with NRP-1 even after 15 minutes or 30 minutes of TGF-β1 stimulation. To further investigate the role of NRP-1 in ENG or ALK1 interaction, we performed ENG and ALK1 staining using colocalization in GAs. There was a minimal colocalization of ENG and ALK1 in GA of *Nrp1^SM22KO^* compared with WT sex-matched littermate animals. Consistent with these results, coimmunoprecipitation studies also showed a reduction of the ENG/ALK1 immune complex in SMCs from *Nrp1^SM22KO^* compared with WT sex-matched littermate animals, suggesting that NRP-1 maintains optimal interactions between ENG and ALK1. In addition, NRP-1 is involved in receptor tyrosine kinase trafficking via direct interaction to elicit maximum signal output in the process (50). These results suggest that NRP-1 deletion in SMCs disrupts ALK/ENG singling, leading to aberrant vascular remodeling and AVMs.

Previous studies have demonstrated that TGF-β1/ALK1/ENG signaling leads to an increase in EC proliferation (51) but not in SMCs (52). Moreover, increased EC proliferation with decreased SMCs is observed in malformed vessels of HHT and in mouse models (15). Consistent with these observations, TGF-β1 stimulation showed a minimal impact on cell proliferation (18% increase) with an increase in cell death, as assessed by increased cleaved caspase-3/7 activity in SMCs isolated from *Nrp1*^SM22KO^ compared with WT sex-matched littermates. There were no changes in cell proliferation from GA from *Nrp1^SM22KO^* compared with WT sex-matched littermate animals, as assessed by Ki-67 staining, with an increase in TUNEL^+^ apoptotic cells located in the media and intima of the artery where SMCs are predominantly located. One plausible explanation for this observation is that NRP-1 could be involved in other downstream signaling pathways, such as PDGF-B, which may compensate for the TGF-β–dependent increased proliferation (29). In addition to that, there was a reduction of SMCs and pericyte lining to the developing vasculature of the retina and adult mouse lungs of *Nrp1^SM22KO^* mice. These results support the notion that *Nrp1* contributes to cell survival and vascular remodeling (53). Although, there are fewer SMCs present in the *Nrp1^SM22KO^* mice compared with WT mice, we could not find a significant difference in apoptotic SMCs index (cleaved caspase-3^+^ cells) in adult lungs of *Nrp1^SM22KO^* mice compared with WT mice (Supplemental Figure 13), and this could be due to efficient clearance of apoptotic cells in the lung (54).

There are limitations to the present study. Though there were enlarged vessels with hemorrhage and increased immune cell infiltration observed in the adult lungs and liver, the vascular permeability with rupture was not assessed. Although histological observations found that there was AVF in the lung, we did not observe an additional capillary bed connection between artery and vein. Finally, this study should be repeated in male mice to see a possible sex hormone influence in the AVMs development.

In conclusion, the present study identified that there is a reduction of NRP-1 in perivascular SMCs in the liver from patients with HHT with *ALK1* mutations. NRP-1 shows a direct biochemical association with ALK1 and ENG, which are upstream receptors to pSMAD1/5/8 signaling, and *Nrp1* ablation attenuates TGF-β1–pSMAD1/5/8 signaling in vascular SMCs. In vivo studies in transgenic adult mice with *Nrp1* deletion in SMCs demonstrate that there is a direct artery-to-vein connection that resembles AVMs in patients with HHT. Moreover, there is dilated vasculature in adult mice with *Nrp1* deletion in SMC lung, liver, and postnatal retinal vessels, along with immune cell infiltration, increased apoptosis, and decreased SMC lining. These results provide evidence for the role of SMC NRP-1 in pathogenesis of vascular malformations.

## Methods

All cell culture supplies were obtained from Thermo Fisher Scientific, unless otherwise specified. All histology supplies except for antibodies were purchased from Dako (Agilent Technologies Inc.).

### Patient tissue samples.

After Mayo Clinic IRB approval (no. 18-004545), we obtained tissue sections from livers of controls and of patients with HHT who required liver transplantation. The patient age and sex are listed in Supplemental Table 1.

### Animal studies.

Mice were maintained under standard conditions of 12-hour/12-hour light/dark cycles, at 22°C and 41% relative humidity with access to food and water ad libitum. We obtained a breeding pair of *Nrp1^fl/fl^* and *SM22α^Cre+^* mice on a C57BL/6J background, and we crossed them to obtain *Nrp1^fl/fl^/*SM22α^Cre+^ (*Nrp1^SM22KO^*) mice as previously described (28, 37). The NRP-1 deletion specific to SMCs was confirmed by performing coimmune fluorescence for SM22 (SMCs), CD31 (27), and NRP-1 staining in lung tissue sections. We also performed Western blot analysis on ECs isolated from the lungs and SMCs isolated from the aorta of *Nrp1^SM22KO^* mice with WT sex-matched littermate.

### Mouse AVF model.

We created an AVF in mice that is seen in HHT AVMs to study hemodynamic changes caused by increased blood flow that occurs in blood vessels of many AVMs (6, 55). An AVF was created by connecting the right carotid artery to the ipsilateral jugular vein in 6- to 8-week-old female mice in *Nrp1^SM22KO^* and WT sex-matched littermate mice as described previously (56–58). The inflow artery supplying blood to the AVF is referred to as GA, and the contralateral left carotid artery is referred to as the CA. Both CA and GA were excised after PBS perfusion at day 3 and day 14 after AVF creation to assess for gene expression and histological changes, respectively.

### Histology.

Paraffin-embedded liver tissue sections from patients with HHT and controls (patients without HHT) were used. Twelve-month-old *Nrp1^SM22KO^* mice with WT sex-matched littermates were euthanized, and lungs were excised after PBS perfusion and then fixed in formalin. Paraffin-embedded tissue blocks were prepared and cut into 5 μm–thick tissue sections. Tissue sections were stained with H&E, VVG (catalog 9116A, Newcomer Supply), Masson’s trichrome staining (catalog 87019, Richard-Allan Scientific), and Carstairs’s staining (catalog 26381, Electron Microscopy Sciences) following the manufactures protocol as described previously (59).

Tissue sections were immunostained after deparaffinization, and heat-induced antigen retrieval was performed in citrate buffer as described (57, 58). Antibody information and sources are listed (Supplemental Table 2). Images were captured using a Zeiss Axio imager M2 equipped with an Axiocam 503 camera and a motorized stage. The intensity of the chromogen stain was quantified using Zen Pro image analysis software, and the percentage of average area of the brown stain to the total tissue area was calculated and presented as stain index (shown as a percentage). ImageJ (NIH) analysis software was used to quantify the fluorescence intensity after immunofluorescence staining. A nonspecific IgG staining was performed to serve as control, and representative images are shown (Supplemental Figure 14, A–C). To assess the area of the pulmonary vasculature and liver vacuoles, we stained every 15th tissue section (typically, there were 60 sections) with CD31. The cross-sectional area of CD31^+^ blood vessels was determined as described previously using Fiji image analysis (60). Briefly, the CD31^+^ vessels in the tissue sections (4 sections per animal) were identified and selected manually using the wand (tracing) tool; then, the area was measured. The percentage of average cross-sectional area/the total tissue area (shown as a percentage) was determined in the lung or liver. We averaged the data for the 4 sections and used the average value for each animal.

### Retinal vascular staining and vascular density measurement.

Eyeballs were removed from P5 mouse pups, and the retinas were dissected as described previously (61). Retinas were then incubated with anti-NG2, and Alexa 594 isolectin-B4 (BSI-B4, catalog I21412, Thermo Fisher Scientific) overnight after permeabilization and blocked with DPBS containing 0.1% Triton X100 and 1% BSA. The retinas were then washed with DPBS containing 0.1% Triton X100 3 times, incubated with Alexa 488 anti–rabbit IgG (catalog 711-545-152, Jackson ImmunoResearch), and flat-mounted and imaged using a fluorescence microscope.

### Gene expression.

RNA was isolated using miRNeasy kit (Qiagen) and cDNA was synthesized using iScript cDNA synthesis kit (Bio-Rad). qPCR was performed using a C1000 thermal cycler with a CFX96 real-time monitoring system using iTaq SYBR green master mix (Bio-Rad). Change in gene expression was calculated following the 2^−ΔΔCT^ method using the TBP-1 as a reference gene. The primer sequences used for the gene expression studies are presented (Supplemental Table 3).

### Isolation of aortic SMCs and pulmonary ECs.

Female littermates of *Nrp1^fl/fl^* (WT) and *Nrp1^SM22KO^* mice of 6–8 weeks old were sacrificed, and the lungs and aorta were immediately removed. SMCs were isolated from pooled aorta (*n* = 3 animals) as described previously (28, 62). For EC isolation, lungs from 3 different animals of the same genotype were pooled and used to isolate cells positive for CD31 and CD102 (ICAM-2) using magnetic beads after collagenase-1 digestion as described previously (63). Isolated SMCs (1 × 10^5^/well) were cultured in a 6-well plate. After overnight serum starvation, cells were stimulated with 10 ng/mL of TGF-β1 (catalog 100-21, PeproTech) for 30 minutes in serum-free DMEM/F12 (64, 65). Cells were lysed in RIPA buffer with inhibitors of proteases (catalog 11836153001, MilliporeSigma) and phosphatases (catalog 4906845001, MilliporeSigma), and protein content was determined using the Bio-Rad DC protein Assay kit (Bio-Rad).

### Cell proliferation and apoptosis.

Aortic SMCs (5000 cells/well) were seeded in a 96-well plate and incubated with 10 ng/mL TGF-β1 overnight, and cell proliferation was assessed using the CellTiter 96 Aqueous One Solution Cell Proliferation Assay (catalog G3580, Promega). Apoptosis was determined based on Caspase-3/7 activity, which was assayed using the Caspase-Glo 3/7 Assay System (catalog G8090, Promega) following the manufacturer’s protocol.

### Coimmunoprecipitation for NRP-1/ALK1 or ENG interactions.

HEK293T cells were cotransfected with plasmids which express NRP-1 and ENG or ALK1 using Lipofectamine-2000. After 48 hours, cells were lysed using IP buffer (50 mM Tris-buffered saline pH 8.0 containing 0.05% sodium deoxycholate, 0.1% NP-40 and inhibitors for proteases and phosphatases). Cell lysates (500 μg) were preclarified with protein A-agarose beads; then, the cell lysates were incubated with 5 μg of NRP-1 antibody at 4°C for overnight. The immune complexes were pulled down with anti–rabbit IgG–coupled magnetic beads and then washed with IP buffer. The immune complexes were extracted from the beads by boiling in Laemmli buffer at 95°C for 5 minutes, and the supernatant was resolved on a 4%–20% SDS-PAGE gel followed by Western blotting. The blots were then probed for anti-FLAG antibody to detect FLAG-tagged ENG or ALK1. For endogenous coimmunoprecipitation, aortic SMCs were isolated from a C57BL/6J mouse and cultured as described above, stimulated with 10 ng/mL of TGF-β1 after overnight serum starvation. Cells were lysed, and immunoprecipitation and Western blot was performed as described above. To test the effect of NRP-1 deletion on ENG/ALK interactions, coimmunoprecipitation experiments were performed with TGF-β1–stimulated cell lysates from WT and *Nrp1^SM22KO^* mice. Cell lysate was incubated with 5 mg of ALK1 antibody and then subjected to Western blotting with an ENG antibody.

### Statistics.

All in vitro cell culture experiments and Western blots were performed in triplicate. Data are expressed as mean ± SEM. For multiple comparisons, significant difference in the mean values were determined by using a 2-way ANOVA. For comparison of 2 groups, significant differences in the mean values were determined using a Mann-Whitney *U* test using Prism 9.0 (Graph Pad Software Inc.). *P* < 0.05 was considered significant.

### Study approval.

All animal experiments were performed after approval was obtained from the Mayo Clinic IACUC (no. 18-004545).

## Author contributions

SK, SM, and DM designed the study. SK and AS performed experiments. DM and YW provided mice. VI, RPG, MST, and SMT provided patient samples and controls. SK, RPG, and SM contributed to histopathology analysis. SK, SM, DM, VI, and YW contributed to data organization and discussion. SK and SM wrote the paper.

## Supplementary Material

Supplemental data

## Version 1 04/05/2022

In-Press Preview

## Version 2 05/09/2022

Electronic publication
