# Supplementary material for: Neuropilin-1 deficiency in vascular smooth muscle cells is associated with hereditary hemorrhagic telangiectasia arteriovenous malformations
Source: JCI Insight. 2022 May 9;7(9):e155565. doi: 10.1172/jci.insight.155565 (PMC9090252; doi:10.1172/jci.insight.155565)
Supplement: Supplemental data [file jciinsight-7-155565-s281.pdf]

# Neuropilin-1 deficiency in vascular smooth muscle cells is associated with Hereditary hemorrhagic telangiectasia arteriovenous malformations

Sreenivasulu Kilari[1], Ying Wang[2,3], Avishek Singh[1], Rondell P Graham[4], Vivek Iyer[5], Scott M Thompson[1], Michael S Torbenson[4], Debabrata Mukhopadhyay[6], Sanjay Misra[1,3*]

[1]Vascular and Interventional Radiology Translational laboratory, Division of Vascular and Interventional Radiology, Department of Radiology; [2] Department of Cardiovascular Medicine; [3]Department of Biochemistry and Molecular Biology, [4]Department of Laboratory Medicine and Pathology, [5]Mayo Clinic Hereditary Hemorrhagic Telangiectasia Center of Excellence, Mayo Clinic, Rochester, Minnesota; [6] Department of Biochemistry and Molecular Biology, College of Medicine and Science, Mayo Clinic, Jacksonville, Florida, United States.

**Running title:** Smooth muscle cell NRP-1 role in AVMs

***Address correspondence to:**

Sanjay Misra, M.D. FSIR, FAHA, Professor of Radiology

Department of Radiology

Mayo Clinic

200 First Street SW

Rochester, MN 55905

Telephone: 507-284-9087

Fax: 507-255-7872

Email: misra.sanjay@mayo.edu

## Supplementary Information:

### Table 1. HHT and control patient demographic information

| Patient/Subject | Age | Sex | Reason for hepatectomy/resection |
|---|---|---|---|
| | | | Controls |
| 1 | 61 | M | Metastatic colon cancer |
| 2 | 43 | M | Metastatic colon cancer |
| 3 | 44 | F | Gallbladder carcinoma |
| 4 | 64 | M | Cholangiocarcinoma |
| 5 | - | | PRC control* |
| 6 | - | | PRC control* |
| | | | HHT |
| 1 | 70 | F | ALK1 mutation |
| 2 | 55 | F | ALK1 mutation |
| 3 | 56 | F | ALK1 mutation |
| 4 | 48 | F | Likely ALK1 mutation (no genetics available) |
| 5 | 47 | F | ALK1 mutation |
| 6 | 47 | F | ALK1 mutation |

***PRC controls:** These samples are being used as standard laboratory control normal liver tissue sections from Mayo Clinic Pathology Research Core (PRC) laboratory and other demographics could not be identified.

## Table 2. Antibodies used for immunostaining and Western blot

| Antibody | Cat# | Source | Concentration |
|---|---|---|---|
| NRP-1 | ab81321 | Abcam | 1:500 (IHC/IF) 5µg (IP*) |
| ALK1 | ab37807 | Abcam | 1:100 (IHC/IF), 5 µg (IP*)<br>1:1000 (Western blot) |
| ENG | AF1320 | R&D systems | 1:100 (IHC/IF)<br>1:1000 (Western blot) |
| FLAG | F3165 | Sigma | 1:500 (Western blot) 5 µg (IP*) |
| SM22α (Transgelin) | SC-53932 | Santa Cruz Biotechnology | 1:500 |
| α−ΣMA | ab7817 | Abcam | 1:500 |
| CD31 | ab28364 | Abcam | 1:1000 (IHC/IF) |
| NG2 | ab5320 | EMD Millipore | 1:500 |
| β-Actin | A1978 | Sigma | 1:5000 |
| Tubulin | T6199 | Sigma | 1:5000 |
| HRP-Anti Rabbit IgG | 7074 | Cell Signaling Technology | 1:1000 |
| HRP-Anti mouse IgG | 7076 | Cell Signaling Technology | 1:1000 |
| Anti-goat IgG HRP | ab97110 | Abcam | 1:1000 |
| Normal Rabbit IgG | SC-2027 | Santa Cruz Biotechnology | 5µg (IP* control) |
| Alexa 488 Anti Rabbit | 711-545-152 | Jackson Immunoresearch | 1:1000 |
| Alexa 488 Anti mouse | 715-545-151 | Jackson Immunoresearch | 1:1000 |
| Alexa 594 Anti Rabbit | 711-585-152 | Jackson Immunoresearch | 1:1000 |
| CD68 | ab125212 | Abcam | 1:1000 (IHC) |
| pSMAD2 | ab53100 | Abcam | 1:100 (IHC) |
| pSMAD3 | ab52903 | Abcam | 1:100 (IHC) |
| SMAD2/3 | 8685 | Cell signaling technology | 1:1000 |
| pSMAD1/5/8 | ab3848-1 | EMD Millipore | 1:100 (IHC) |
| pSMAD1/5/8 | 11971S | Cell signaling technology | 1:1000 (Western Blot) |
| TGF-b1 | SC 146 | Santacruz | 1:400 |
| TNF-α | C10265 | Assay biotech | 1:50 |
| CD31 | 550274 | BD Bioscience | Endothelial cells isolation |
| ICAM-2 | 553326 | BD Bioscience | Endothelial cells isolation<br>Western blot (1:1000) |
| Anti-Rat IgG- magnetic beads | S1433 | New England biolabs | Endothelial cell sorting |

## Table 3. Mouse primer sequences used for qRT-PCR gene expression analysis

| Gene | Forward (5'-3') | Reverse (5'-3') |
|---|---|---|
| *NRP-1* | *TCCTGGGAAACTGGTATATCTATGA* | *CATTCCAGAGCAAGGATAATCTG* |
| *TGF-β1* | *CCTGAGTGGCTGTCTTTTGA* | *TCGTGGAGTTTGTTATCTTTGCTG* |
| *TNF-α* | *GCTCTTCTGTCTACTGAACTTCG* | *GATGAGAGGGAGGCCATTTG* |
| *ALK1* | *GGCCTTTTGATGCTGTCG* | *ATGACCCCTGGCAGAATG* |
| *ENG* | *TACCTCTGGATACCGGATAAGG* | *GTAGATCACAGCCGACTCTTTC* |
| *TBP-1* | *AAGGGAGAATCATGGACCAG* | *CCGTAAGGCATCATTGGACT* |
| *BMP9* | *CAACAGATACACAACGGACAAATC* | *CTCGGGATGGAGATGTTGAAG* |
| *SMAD6* | *ATTCTCGGCTGTCTCCTCCT* | *CCCTGAGGTAGGTCGTAGAA* |
| *SMAD7* | *AGGCTGTGTTGCTGTGAA* | *CCATTGGGTATCTGGAGTAAGG* |

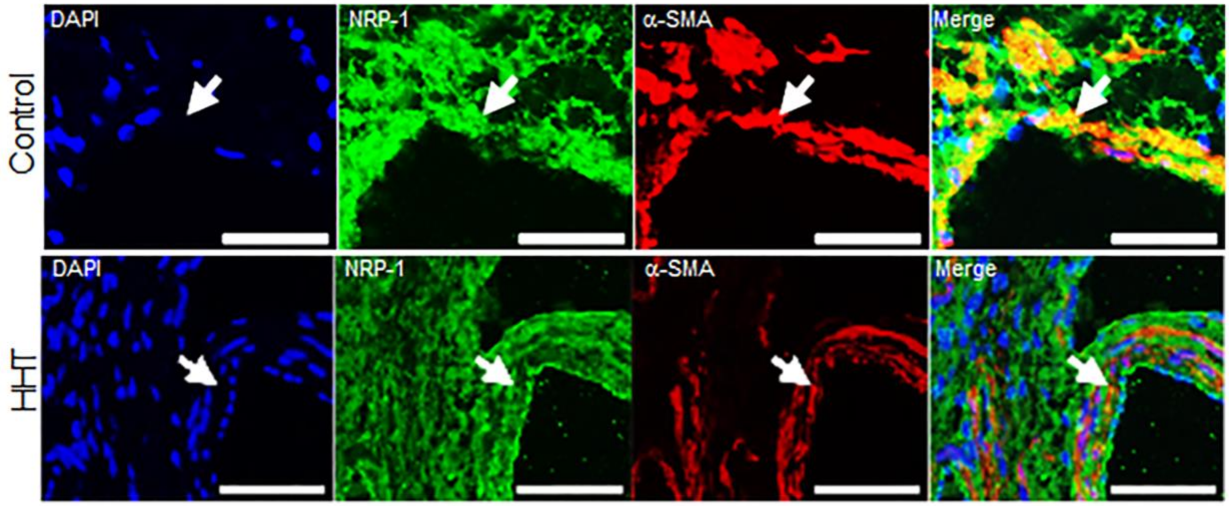


**Figure 1.** Co-immune staining for individual color channels from NRP-1 (green), α-SMA (Red), and DAPI stained nucleus (Blue) of liver sections from HHT patients with controls. All images were captured at 10X magnification using a Zeiss Axio imager M2 equipped with an Axiocam 503 camera and a motorized stage. Scale bar is 50-µm.

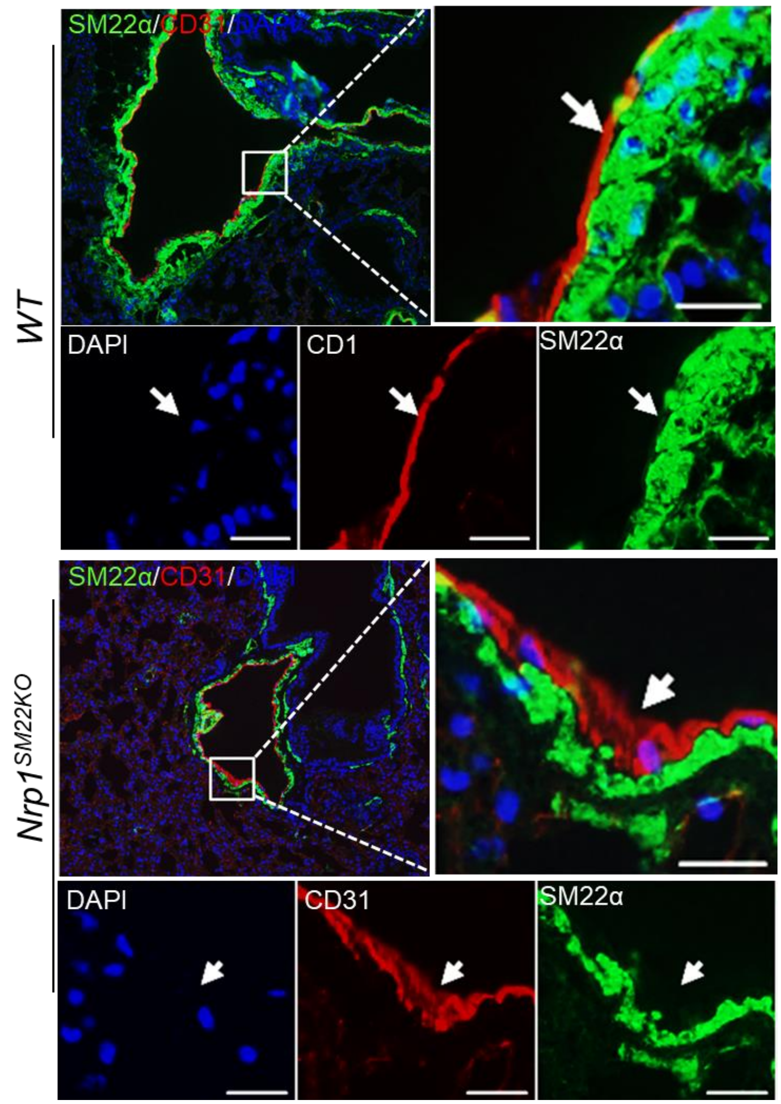


**Figure 2.** Co-immune staining for CD31 (red), SM22α (green) and DAPI (blue) stained nucleus in representative images of lungs from *Nrp-1*$^{f/f}$ (*WT*) and *Nrp-1*$^{f/f}$*/SM22a*$^{Cre+}$ (*Nrp-1*$^{SM22KO}$) adult mice are shown as separate panels. Arrow heads indicate cells positive for CD31. There is an absence of SM22α (green) stain in CD31(+) cells stained red in both *WT* and *Nrp1*$^{SM22KO}$ adult mouse lungs. All images were captured at 10X magnification. Scale bar is 20-µm.

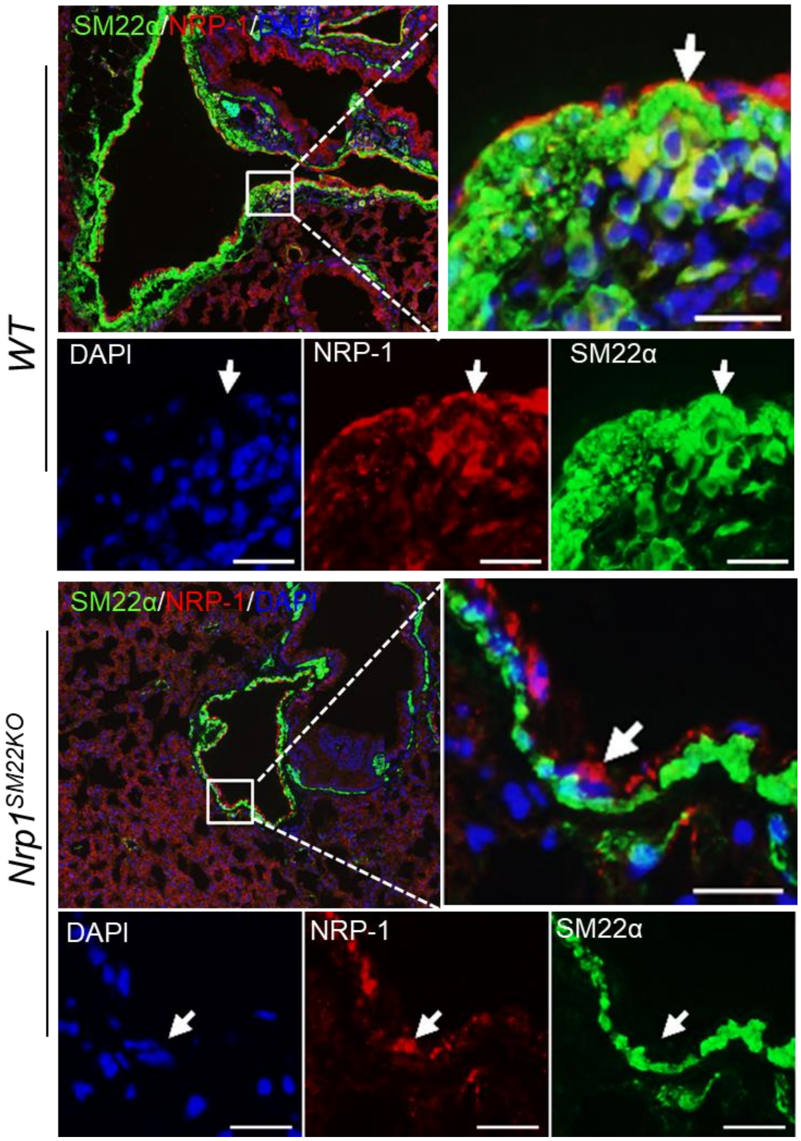


**Figure 3.** Co-immune staining for NRP-1 (Red), SM22 staining (Green) and DAPI (Blue) stained nucleus in representative images are shown as separate panels. Arrow heads indicate cells positive for NRP-1. There is an absence of NRP-1 in SM22 (+) cells in mouse lungs from *NRP-1*$^{SM22KO}$ and *WT* sex-matched littermates. All images were captured at 10X magnification using a Zeiss Axio imager M2 equipped with an Axiocam 503 camera and a motorized stage. Scale bar is 20-μm.

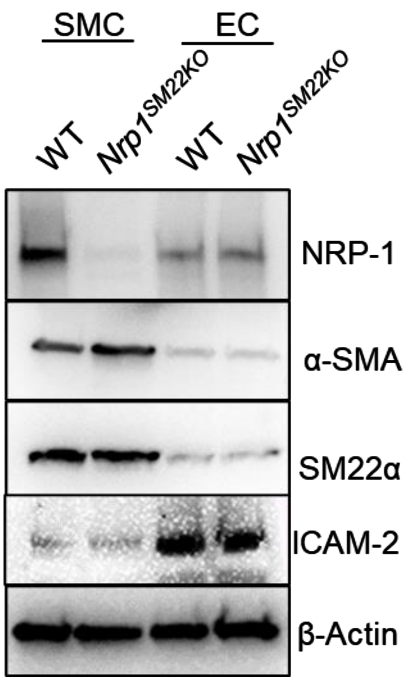


**Figure 4.** NRP-1 protein expression in pooled smooth muscle cells (SMC) and endothelial cells (EC) from three different animals. Aortic SMCs and pulmonary ECs were isolated as described and Western blotting was performed to assess for NRP-1 using markers for SMCs (α-SMA) and ECs (ICAM-2). There is absence of NRP-1 in SMCs with no difference in NRP-1 expression in ECs from *Nrp1$^{SM22KO}$* mice compared to *WT* sex-matched littermates animals indicating NRP-1 deletion is restricted to SMCs. There was a minimal expression of SM22 and α-SMA in ECs compared to SMCs.

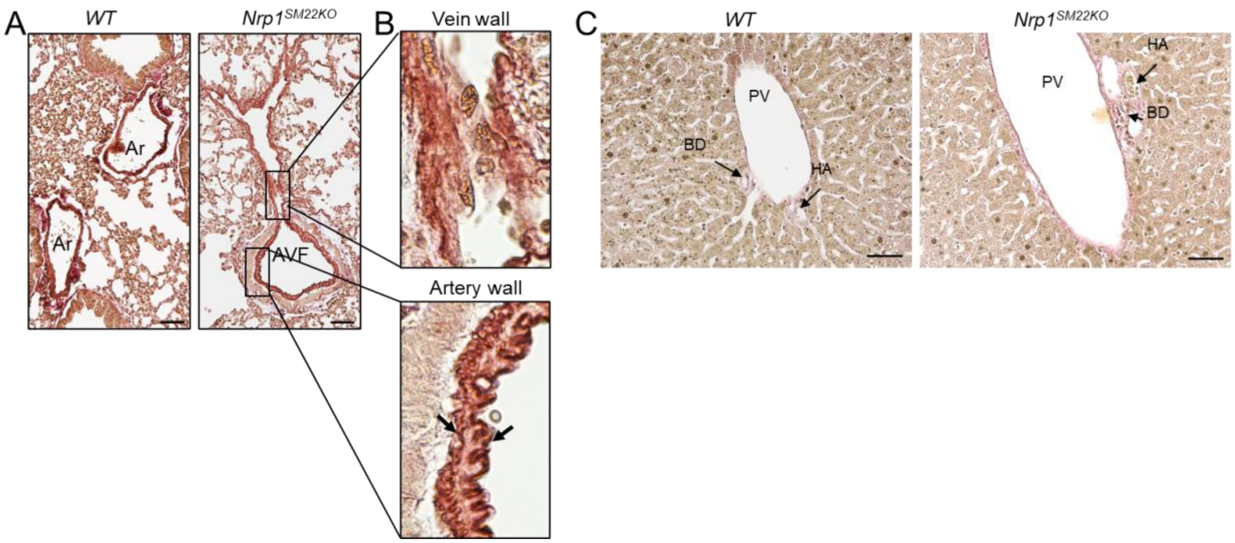


**Figure 5.** Verhoeff-Van Gieson (VVG) staining of lungs and livers from *Nrp-1*$^{f/f}$ (*WT*) and *Nrp-1*$^{f/f}$*/SM22a*$^{Cre+}$ (*Nrp-1*$^{SM22KO}$) adult mice**.** VVG staining was performed to identify the artery and vein in the (A-B) lung and (C) liver. (B) The areas enclosed with boxes in A are digitally enlarged and shown as separate panels. Artery shows both an external and internal elastin lamina as indicated by arrowheads and vein shows an internal elastin lamina only. (A-B) Ar; artery, and AVF; an arteriovenous fistula of artery to vein direct connection. (C) BD; bile duct, PV; portal vein and HA; hepatic artery. All images were captured at 10X magnification. Scale bar is 50-µm.

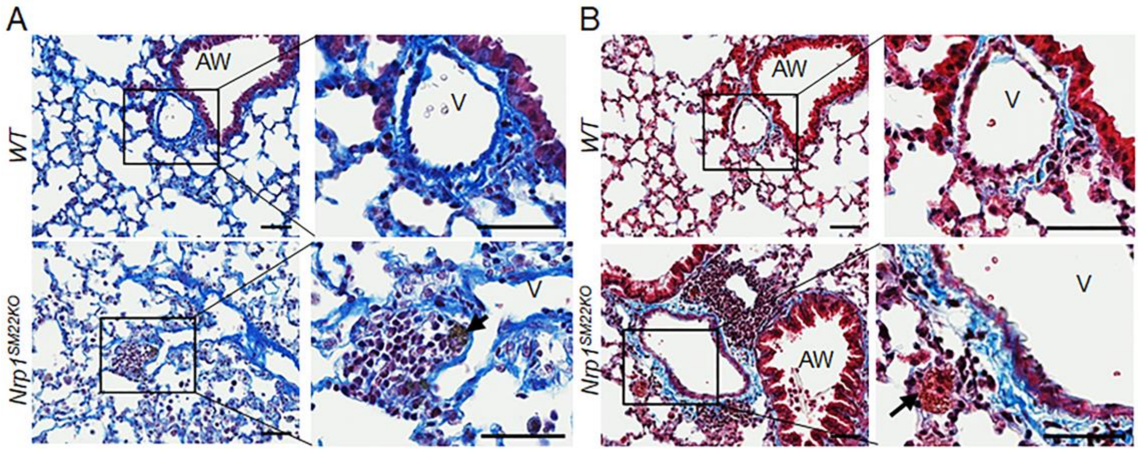


**Figure 6.** Carstairs’s and Masson’s trichrome staining of mouse lungs shows extravascular presence of blood of *Nrp1*$^{SM22KO}$ mice compared to *WT* sex-matched littermates. (A) Carstairs's stain identifies collagen (bright blue), muscle (red), platelets (gray blue/navy), red blood cells (yellow), and fibrin (bright red). (B) Masson’s trichrome stain shows collagen (blue), cytoplasm, muscle fibers (red), blood cells (yellow/red), and nuclei (black/blue). Arrow heads indicate the presence of extravascular red blood cells. AW; airway (thick walls with red muscle tissue) and V; blood vessel. All images were captured at 10X magnification using a Zeiss Axio imager M2 equipped with an Axiocam 503 camera. Scale bar is 50-µm.

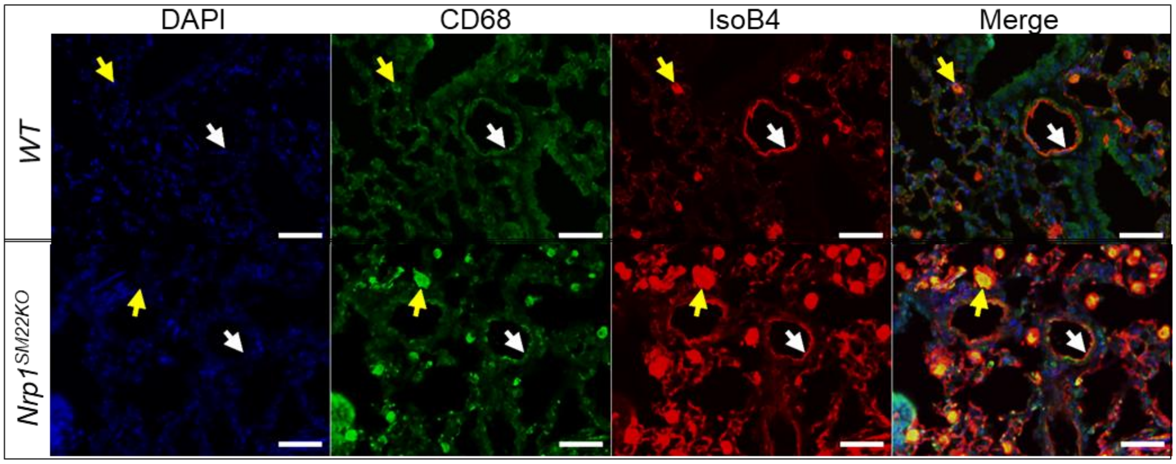


**Figure 7.** CD68 (+) cell infiltration in lung interstitial space of *Nrp1*$^{SM22KO}$ mice compared to *WT* sex-matched littermates. Co-immune staining for CD68 (green) and isolectin-B4 staining (Red) from representative images are shown as separate panels. White arrow heads indicate isolectin-B4 positive endothelium (Red), and yellow arrow heads indicate CD68 (+) cells which also co-stained for isolectin-B4. All images were captured at 10X magnification using a Zeiss Axio imager M2 equipped with an Axiocam 503 camera and a motorized stage. Scale bar is 50-μm.

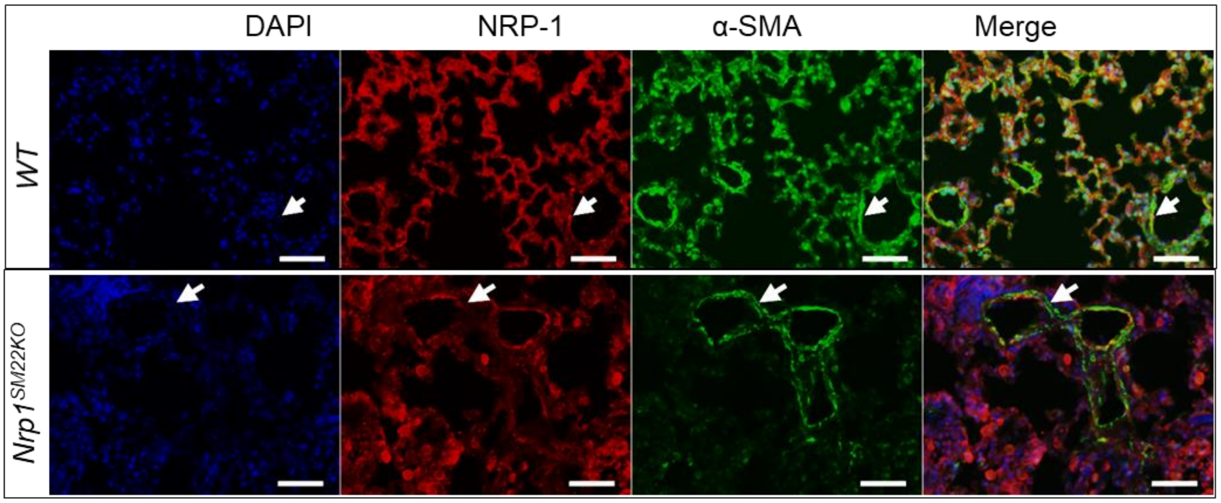


**Figure 8.** Co-immune staining for NRP-1(Red), α-SMA staining (Green) and DAPI (Blue) stained nucleus of *Nrp1*$^{SM22KO}$ mice compared to *WT* sex-matched littermates in representative images are shown as separate panels. Arrow heads indicate cells positive for both α-SMA and NRP-1. All images were captured at 10X magnification using a Zeiss Axio imager M2 equipped with an Axiocam 503 camera and a motorized stage. Scale bar is 50-μm.

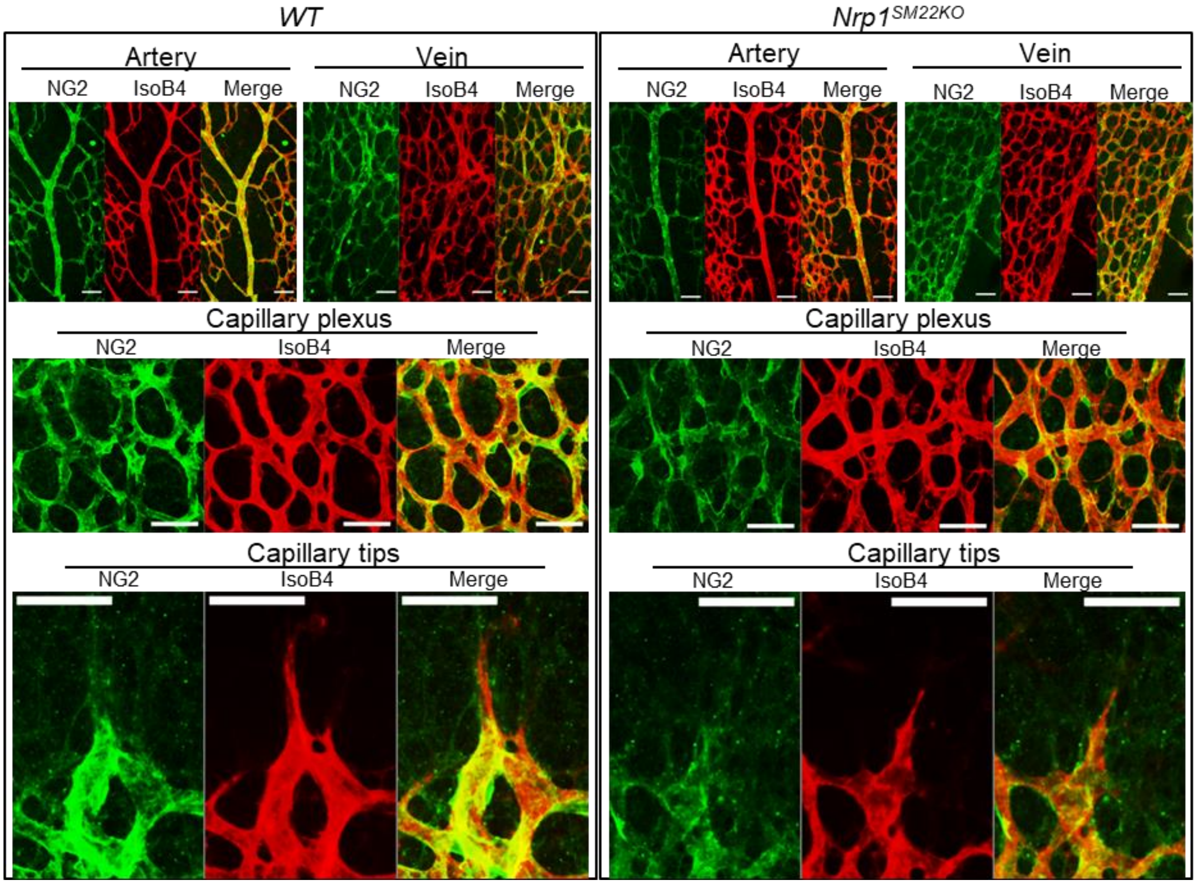


**Figure 9.** Representative images of postnatal day 5 retina from *Nrp1*$^{SM22KO}$ mice compared to *WT* sex-matched littermates mice pups were co-stained for red-isolectin-B4 (IsoB4) and green-NG2 for pericytes and shown as individual channels. All images were captured at 10X magnification using a Zeiss Axio imager M2 equipped with an Axiocam 503 camera and a motorized stage. Scale bar is 50-µm.

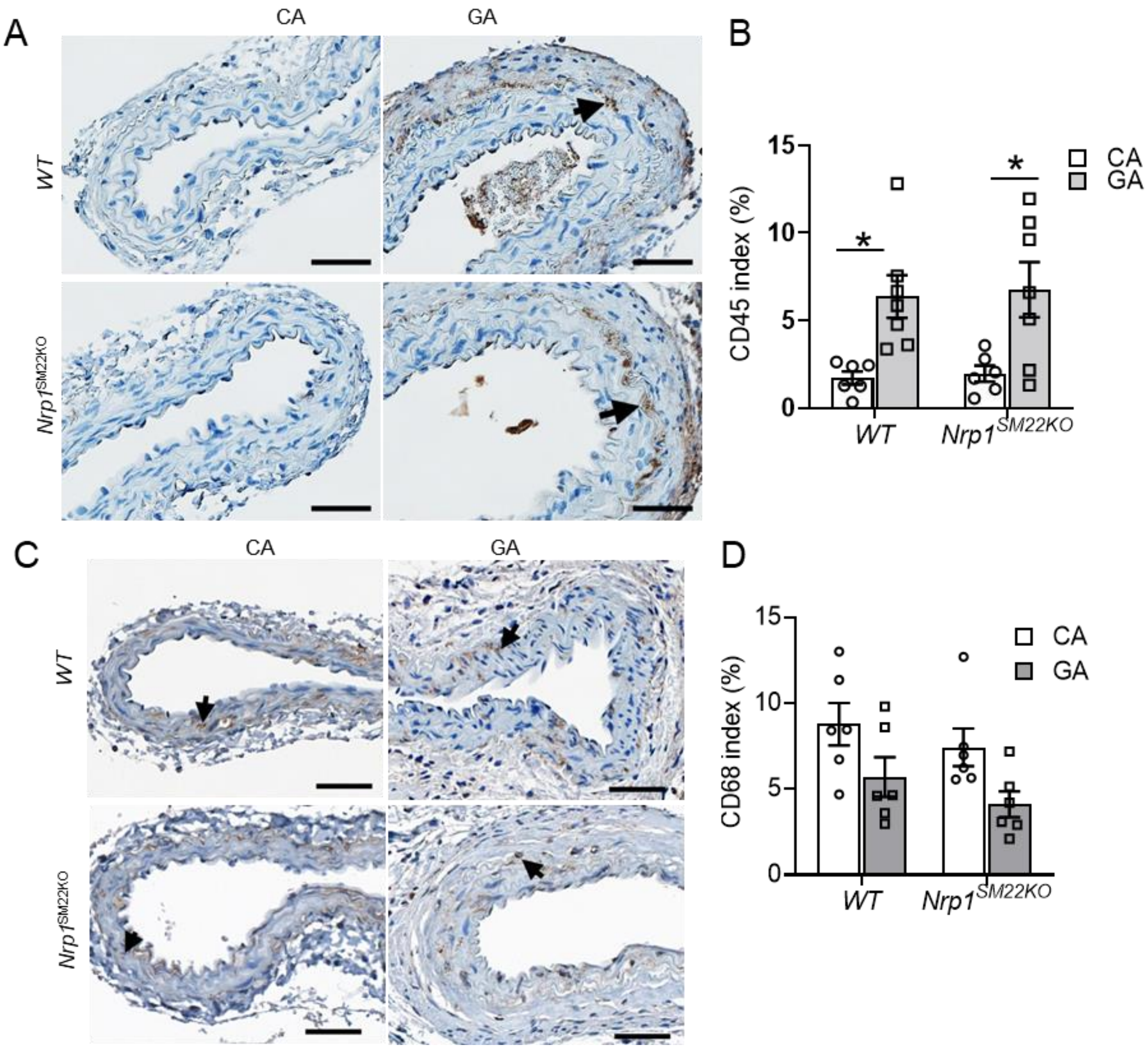


**Figure 10.** There is no significant difference in CD68 and CD45 positive cells in mouse AVF inflow arteries (GA) of *Nrp1*$^{SM22KO}$ mice compared to *WT* sex-matched littermates. Immunostaining was performed for CD68 and CD45 to determine immune cell infiltration at 14 days after AVF creation in the inflow arteries. Representative images of (**A**) CD45 and (**C**) CD68 stained tissue sections of contralateral carotid artery (CA) and AVF inflow artery (GA). Scale bar is 50-µm. The arrow heads indicate in (A) CD45 (+) cells and (B) CD68 (+) cells in brown stain. All images were captured at 10X magnification using a Zeiss Axio imager M2 equipped with an Axiocam 503 camera and a motorized stage. The percentage of tissue area positive for brown stain

was measured using Zen Pro image analysis software (Zeiss). Each data point in the bar graphs represents the mean ± SEM of the (B) CD 68 and (D) CD 45 indices with n of 6. *P<0.05.

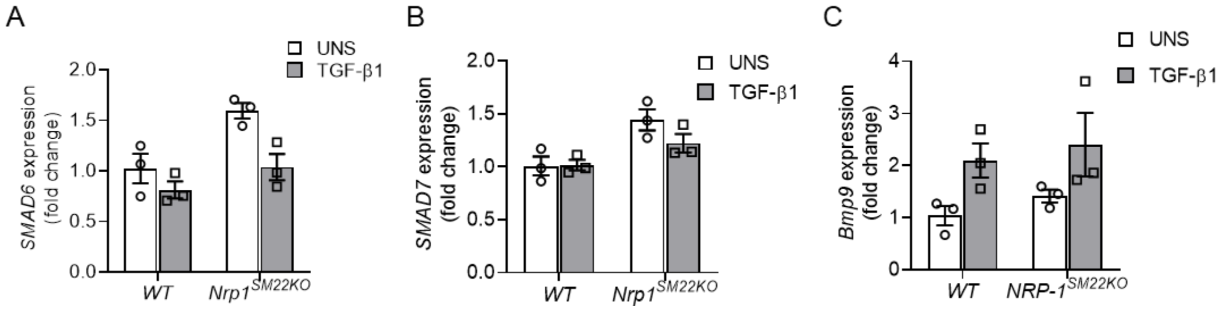


**Figure 11.** TGF-β1 stimulation had no effect on gene expression of *SMAD6*, *SMAD7* and *BMP9* in SMCs from aorta of *Nrp1*$^{SM22KO}$ mice compared to *WT* sex-matched littermates. Gene expression was assessed using qRT-PCR in SMCs from aorta. After overnight serum starvation, SMCs were stimulated with 10 ng/ml TGF-β1 for 24h and gene expression was assessed as described. Gene expression of (A) *SMAD6*, (B) *SMAD7* and (C) *BMP9* in SMCs with (TGF-β1) and without (UNS) TGF-β1 stimulation. The data are normalized to the gene expression in unstimulated (UNS) SMCs from *WT* mouse and expressed as mean fold change ± SEM (n =3). A two-way ANOVA followed by Student *t*-test was performed with Bonferroni's correction. * indicates $P<0.05$.

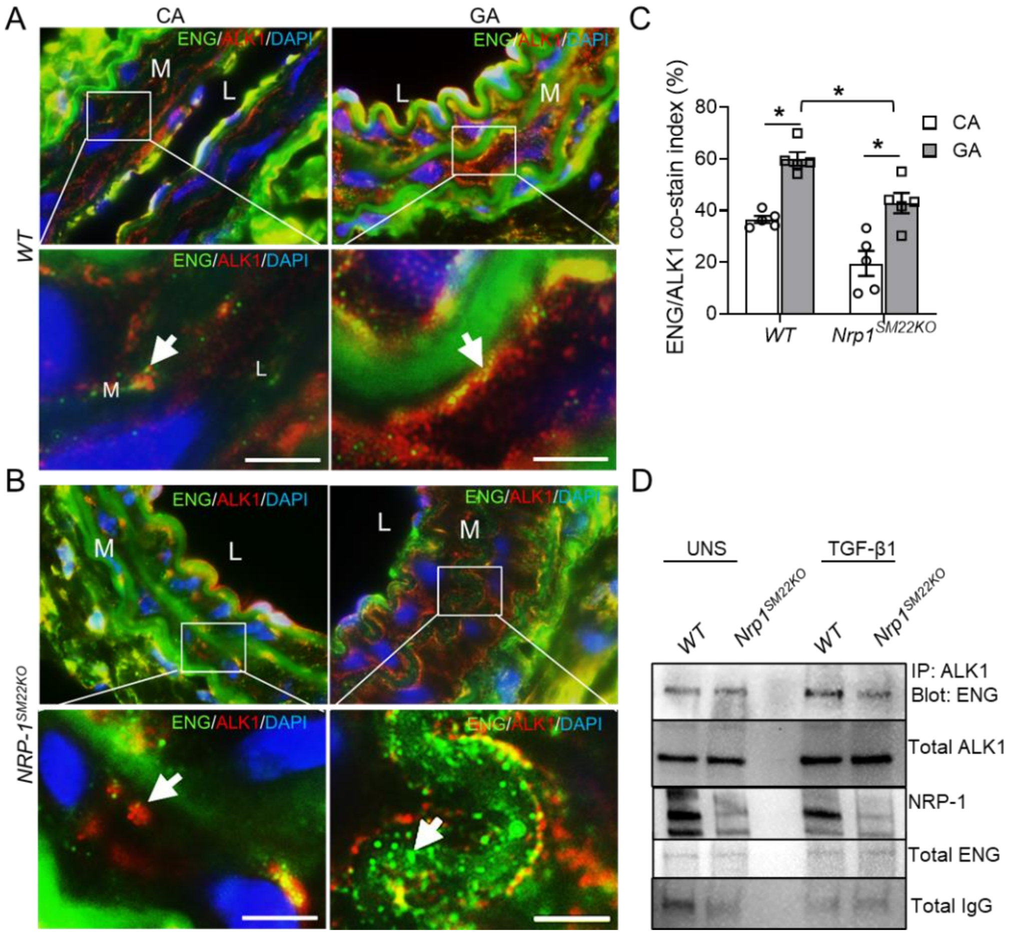


**Figure 12.** There is decreased biochemical interaction between ENG and ALK1 in SMCs from *Nrp1*$^{SM22KO}$ mice compared to *WT* sex-matched littermates. Representative images of AVF inflow arteries from (A) WT and (B) *Nrp1*$^{SM22KO}$ animals co-immune stained for ALK1 (Red), ENG staining (Green), and DAPI (Blue) stained nucleus are shown. Arrow heads indicate ALK1 and ENG (+) cells. All images were captured at 100X magnification using a Zeiss Axio imager M2 equipped with an Axiocam 503 camera and a motorized stage and images in the upper panels were digitally enlarged and shown as lower panels. Scale bar is 5-µm. (C) Co-localization of ENG and ALK1 were quantified by using NIH-Image J software with co-localization analysis plugin. Each data point in the bar graphs represents the mean ± SEM of n=6. A two-way ANOVA followed by Student *t*-test was performed. * indicates P<0.05. (D) ENG is co-immunoprecipitated with

endogenous ALK1 in mouse aortic SMCs. TGF-β1 stimulation increase ENG association with ALK1 in SMCs compared to unstimulated cells (UNS) from WT mice compared to *Nrp1*$^{SM22KO}$ mice.

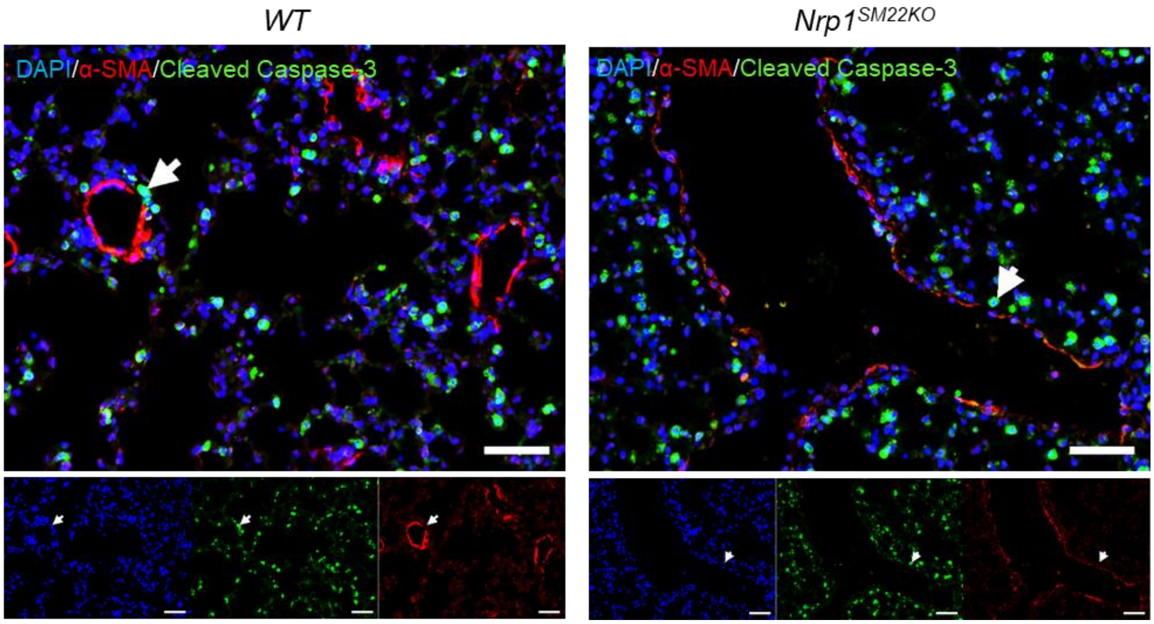


**Figure 13.** Representative images of lungs from *Nrp1*$^{SM22KO}$ mice compared to *WT* sex-matched littermates have been co-immune stained for α-SMA (Red), cleaved caspase-3 (Green), and DAPI (Blue) are shown. Arrow heads indicate cells positive for cleaved caspase-3. There was no co-staining of α-SMA (red) and cleaved caspase-3 (green) in *WT* and *Nrp1*$^{SM22KO}$ animals (n=5). All images were captured at 20X magnification using a Zeiss Axio imager M2 equipped with an Axiocam 503 camera. Individual color channels are shown in the lower panel. Scale bar is 50-µm.

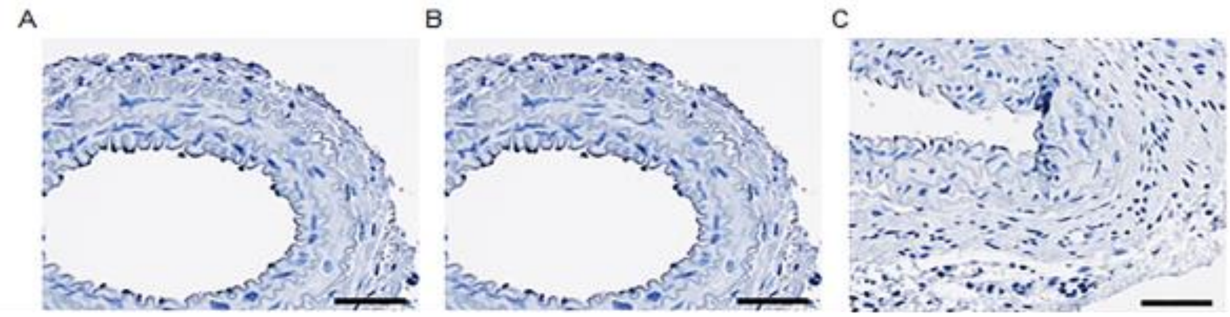


**Figure 14.** Nonspecific IgG control staining of mouse AVF inflow arteries. Immunostaining was performed in mouse AVF inflow artery with normal rabbit IgG (A), normal mouse IgG (B) and normal goat IgG (C) followed by DAB chromogen staining. The absence of brown staining indicates that there is lack of non-specific secondary antibody binding to the tissue section. All images were captured at 10X magnification using a Zeiss Axio imager M2 equipped with an Axiocam 503 camera. Scale bar is 50-µm.
